# Rates of Depression in Children and Adolescents With ADHD: A Systematic Review and Meta-Analysis

**DOI:** 10.1177/10870547251341597

**Published:** 2025-06-27

**Authors:** Shipei Wang, Tracy M. Stewart, Isinsu Ozen, Arnab Mukherjee, Sinead M. Rhodes

**Affiliations:** 1 Centre for Clinical Brain Sciences, University of Edinburgh, Edinburgh, UK; 2 Child Life and Health, University of Edinburgh, Edinburgh, UK; 3 Moray House School of Education and Sport, University of Edinburgh, Edinburgh, UK; 4 Cambridgeshire and Peterborough NHS Foundation Trust, UK; 5 General Adult Psychiatry, Inverclyde Royal Hospital, NHS Greater Glasgow & Clyde, UK

**Keywords:** ADHD, depression, rate, co-occurrence, children, adolescents

## Abstract

**Objective:**

Accumulating evidence indicates high rates of major depressive disorder (MDD) in children and adolescents with ADHD. This systematic review and meta-analysis aimed to examine the rate of depression in children and adolescents with ADHD who are without intellectual disability (ID).

**Method:**

A comprehensive search of six databases identified 20,745 studies. After screening based on inclusion and exclusion criteria, 24 studies were retained. A meta-analysis estimated the pooled depression rate in this population, and subgroup analyses examined differences based on sex, pubertal status, ADHD medication use, recruitment settings, depression assessment tools, informants, and risk of bias rating. Depression rates in children and adolescents with ADHD were compared with neurotypical peers in the retained case-control studies.

**Results:**

Depression rates in children and adolescents with ADHD across the included studies ranged from 1.7% to 60%, with the meta-analysis estimating a pooled depression rate of 11.31% (95% CI [0.07, 0.16]). Subgroup analyses indicated significant differences by sex, with females showing higher rates than males. Differences were also noted by assessment methods, with the highest rates observed when both questionnaires and interviews were used. While other factors did not significantly affect rates, notable trends were identified and reported in the current article.

**Conclusion:**

Depression is a common co-occurrent psychiatric condition in children and adolescents with ADHD, with rates observed in this review and meta-analysis being higher than those reported for neurotypical children and adolescents. This review underscores the importance of combining multiple assessment methods to capture a comprehensive picture of depression in this population, as well as ensuring balanced demographic representation. This review also suggests that further research should explore the depression developmental patterns in children and adolescents with ADHD and identify whether patterns are similar to the neurotypical population.

## Introduction

ADHD is a neurodevelopmental disorder characterized by symptoms including excessive activities, difficulty staying still for long periods, and difficulties in cognitive function (American Psychiatric Association, 2013). ADHD affects over 5% of the pediatric population worldwide (Barkley, 2015; Polanczyk et al., 2014). Researchers, clinical practitioners, and those with lived experiences of ADHD increasingly emphasize the important influence of co-occurring mental health conditions on the health, well-being, and mortality rates of those with ADHD (Meinzer & Chronis-Tuscano, 2017; Thapar et al., 2023). It is estimated that about 55% to 65% of children and adolescents aged 4 to 15 years with ADHD are diagnosed with at least one psychiatric condition other than ADHD, and over 30% are diagnosed with two or more (Cuffe et al., 2020), markedly higher than observed in neurotypical young people (Patel et al., 2007). Among these co-occurring psychiatric conditions, depression is common in ADHD (Daviss, 2008; Meinzer et al., 2014).

Depression is a heterogeneous mood disorder, characterized by symptoms such as low mood or anhedonia, sleep disturbances, fatigue, weight loss/gain, cognitive difficulties, psychomotor changes, worthlessness, and suicidal ideation (American Psychiatric Association, 2013). In the neurotypical population, depression prevalence remains relatively low before early adolescence (Kessler et al., 2001; Maughan et al., 2013; Thapar et al., 2012); this prevalence steadily increases from early adolescence to approximately 6% during the middle to late stages of adolescence (Costello et al., 2006, 2011). The upward trend in depression prevalence accelerates significantly from middle to late adolescence in this population, with a considerable sixfold increase between 15 and 18 years old (Hankin & Abela, 2005; Hankin et al., 1998). Depression is recognized as a risk factor for maladaptive outcomes in the neurotypical population, including increased rates of substance abuse, suicide, intimate partner violence victimization, and a higher prevalence of mental health conditions (Johnson et al., 2018; McLeod et al., 2016). Co-occurring ADHD and depression can exacerbate challenges (Biederman et al., 2008; Meinzer et al., 2014). For instance, a literature review reported that adolescents with co-occurring ADHD and depression have a higher risk of long-term impairment in social functioning and suicide compared to those diagnosed with either condition alone (Daviss, 2008). Supporting this, Zahid et al. (2020) found that adolescents with co-occurring ADHD and major depressive disorder (MDD) had a 52.2% increased risk of suicide than those with MDD alone.

One systematic review has examined the prevalence of co-occurring psychiatric conditions in adults with ADHD, reporting a heterogeneity in the prevalence of depression, ranging from 8.6% to 55% (Choi et al., 2022). However, no systematic review has specifically focused on rates of depression in children and adolescents (≤18 years old) with ADHD. Co-occurring psychiatric conditions during childhood and adolescence may exacerbate ADHD symptoms, contribute to greater cognitive functioning difficulties, reduce the effectiveness of interventions and treatments, and result in a poorer overall prognosis for children and adolescents (Antshel et al., 2016; Ashwood et al., 2015). Thus, it is important to examine co-occurring depression rates in children and adolescents with ADHD (Hartman, 2023). Research has suggested that children and adolescents with ADHD may have different depressive characteristics than their neurotypical peers. Specifically, anhedonia, which refers to the loss of interest and pleasure in daily activities (American Psychiatric Association, 2013), emerges as the strongest predictor of depression in children and adolescents with ADHD (Babinski et al., 2019), whereas the most predictive symptom in neurotypical children and adolescents is low mood or irritability (Luby et al., 2009). Given these differences, understanding rates of co-occurring depression in children and adolescents with ADHD would be beneficial, especially for tailoring interventions and improving outcomes (Turgay & Ansari, 2006).

Various factors may influence depression rates in children and adolescents with ADHD. Similar to findings in the neurotypical population (e.g., Joinson et al., 2012; Stumper & Alloy, 2023), previous research has shown that adolescents with ADHD in the later stages of puberty have higher levels of depression compared to those at earlier stages (Eng et al., 2023). This suggests that variations in participants’ pubertal status may contribute to reported heterogeneity in depression rates. Another potential factor is sex. While some studies have reported no sex differences in depression among children and adolescents with ADHD (e.g., Lahey et al., 2007; Yoshimasu et al., 2012); others have shown that female adolescents and adults with ADHD have a significantly higher depression incidence compared to their male counterparts (e.g., Rucklidge, 2008; Solberg et al., 2018), consistent with findings observed in neurotypical populations (e.g., Salk et al., 2017; Shorey et al., 2022).

Empirical research findings on the impact of ADHD medications (e.g., methylphenidate [MPH]) on depression in children and adolescents with ADHD are mixed. Some research has found that the use of MPH can reduce the incidence of depression in children, adolescents, and adults with ADHD (Chang et al., 2016); while others indicate that children and adolescents with ADHD experienced increased levels of depression during MPH treatment (Oh et al., 2022). Thus, ADHD medication use is another important factor to consider when investigating depression rates in this population. Differences in recruitment pathways when investigating depression rates in ADHD may also contribute to heterogeneity reported in the literature. In comparison to those recruited through community pathways, children and adolescents with ADHD recruited through clinical pathways have been shown to experience more difficulties in interpersonal, self-actualization, and adaptive functioning (Bauermeister et al., 2007) and are more likely to present with co-occurring psychiatric conditions, including depression (Compas et al., 1997; Goodman et al., 1997).

The lack of psychometrically validated depression measurement tools specifically designed for children and adolescents with ADHD likely contributes to heterogeneity in depression rates reported in the literature. For example, the overlap between ADHD and depression symptoms (e.g., inattention and irritability) may cause children and adolescents with ADHD, as well as their parents, to inaccurately report elevated depression scores on scales originally designed and standardized for the neurotypical population (Davidsson et al., 2017; Lundervold et al., 2016). Moreover, some children and adolescents with ADHD may face working memory difficulties (Kanevski et al., 2023; Rhodes et al., 2012), which may affect their reading comprehension and interpretation of written language (Kofler et al., 2019). These challenges may introduce variability in how children and adolescents with ADHD interpret and respond to depression measures designed for the neurotypical population. Informant type for depression assessment may also influence reported depression rates, as relying solely on either children or parents may reflect differing perspectives and interpretations (De Los Reyes & Kazdin, 2005). For example, children with ADHD often report lower depression levels compared to their parents (Fraser et al., 2018). These potential differences suggest the need to consider informant type as a factor that contributes to heterogeneity in reported depression rates in the ADHD population.

It is vital to better understand rates of depression in ADHD as it can help shape healthcare policies, facilitate targeted interventions, and provide appropriate support services (Johansson et al., 2013; Williams et al., 2009); it can also contribute to advancing research on the co-occurrence of ADHD and depression (Meinzer et al., 2014). Despite this importance, there have been no systematic reviews and meta-analyses to date estimating rates of depression in children and adolescents with ADHD. This systematic review and meta-analysis aimed to identify rates of MDD in children and adolescents with ADHD, whether MDD is diagnosed clinically or identified through clinical cut-off scores on standardized questionnaires. This systematic review also aimed to examine whether rates differ by sex, pubertal status, ADHD medication use, recruitment methods, depression assessment tools, and informants for depression assessment. Based on these findings, the review made recommendations for future studies.

## Methodology

### Reporting

This review adhered to the *Preferred Reporting Items for Systematic Reviews and Meta-Analyses* (PRISMA) guidelines (Moher et al., 2009). The protocol of this review was registered on PROSPERO (CRD42023418062).

### Search Strategy

Common synonyms and medical subject headings (MESH) for keywords were developed and adapted from previous systematic reviews on “depression” (Lima et al., 2013; Stewart et al., 2022), “ADHD” (Eaton et al., 2023; McDougal et al., 2022), and “children/adolescents” (Eaton et al., 2023; Stewart et al., 2022; Vacher et al., 2020). Search keywords are detailed in Table 1. Multiple keywords from different categories are combined using the Boolean operator “AND,” and multiple keywords from the same category are connected using the Boolean operator “OR” (Sayers, 2008).

**Table 1.** Keywords and Combinations Used in the Search Strategy.

	Search Keywords
S1	“Attention Deficit Hyperactivity Disorder” OR “Attention deficit disorder with hyperactivity” OR “attention-deficit/hyperactivity disorder” OR “ADHD” OR “ADD” OR “attentional deficits” OR “attentional disorder” OR “attention deficit disorder” OR “hyperkinetic disorder” OR “hyperkinetic syndrome”
S2	“depress*” OR “major depressive disorder*” OR “major depress*” OR “depressive disorder*” OR “MDD” OR “mood disorder*” OR “affective disorder*” OR “clinical depress*”
S3	“child*” OR “adoles*” OR “youth*” OR “young*” OR “teen*” OR “minor*” OR “boy*” OR “girl*”
S4	S1 AND S2 AND S3

The keywords shown in Table 1 were used to search in six databases, including EMBASE, ERIC, PsycINFO, CINAHL, MEDLINE, and Web of Science. The search included research from 1992 to August 2023 based on the ADHD diagnostic criteria of the International Classification of Diseases 10th Revision (ICD-10) and later versions (e.g., ICD-11), or studies from 1994 to August 2023 based on the ADHD diagnostic criteria of the Diagnostic and Statistical Manual of Mental Disorders Fourth Edition (DSM-IV) and its subsequent versions (e.g., DSM-IV-TR and DSM-5). ICD-10, published in 1992, was the first to divide ADHD subtype diagnoses, including inattentive, hyperactive-impulsive, and combined type (World Health Organization, 1992); while DSM-IV, published in 1994, adopted nearly identical criteria to classify these ADHD subtypes (American Psychiatric Association, 1994). The search was conducted on 31st August 2023 and focused exclusively on studies involving human participants that were published in peer-reviewed journals in English. Any duplicate records found during the search were removed from the results of included studies.

### Selection, Inclusion, and Exclusion

This systematic review included empirical studies involving children and adolescents ≤18 years old (i.e., studies with some participants over 18 years were retained if the mean sample age was ≤18). Inclusion criteria required that ADHD be clinically diagnosed according to the DSM-IV (or DSM-IV-TR/DSM-5) or ICD-10 (or ICD-11) criteria, or confirmed using a validated diagnostic tool (e.g., Kindle Schedule for Affective Disorders and Schizophrenia for School-Age Children [K-SADS]); Studies were eligible for inclusion if they utilized a specific depression measure, a depression subscale within a generic instrument, or assessed depression based on DSM or ICD. This review focused exclusively on children and adolescents with ADHD but without Intellectual Disability (ID) to minimize the potential confounding effects of low IQ on rates of depression. This focus was chosen because there is evidence that depression severity differs between children and adolescents who have both ADHD and ID and those with ADHD but without ID, with children and adolescents with co-occurring ADHD and ID tending to report higher levels of depression (Ahuja et al., 2013; Pearson et al., 2000). In this review, participants were defined as having no ID if they had a full-scale IQ (FSIQ) of 70 or higher (American Psychiatric Association, 2013).

Studies that did not meet the inclusion criteria were excluded. Specifically, studies were excluded if the mean age of participants exceeds 18 years, if ADHD was diagnosed using criteria predating DSM-IV/ICD-10, if only screening tools (e.g., ADHD Rating Scale [ARS] and Turgay DSM-IV Disruptive Behavior Disorders Rating Scale-Teacher and Parent Forms [T-DSM-IV-S]) were used, or if the ADHD diagnostic procedure was not reported. Additionally, studies focusing on sub-clinical depression (i.e., below the clinical threshold) or examining the lifetime rates of depression, rather than current rates, were also excluded. Theses, systematic reviews, meta-analyses, literature reviews, book chapters, or conference proceedings/abstracts were also excluded. Intervention studies were excluded, as were longitudinal studies that did not provide baseline or follow-up depression assessment data in participants. Studies were also excluded if they employed measures that did not assess depression separately. For example, if a study employed a scale that measured internalizing symptoms, that is a joint measure of depression and anxiety without reporting the two separately, it was excluded.

**Figure 1.**
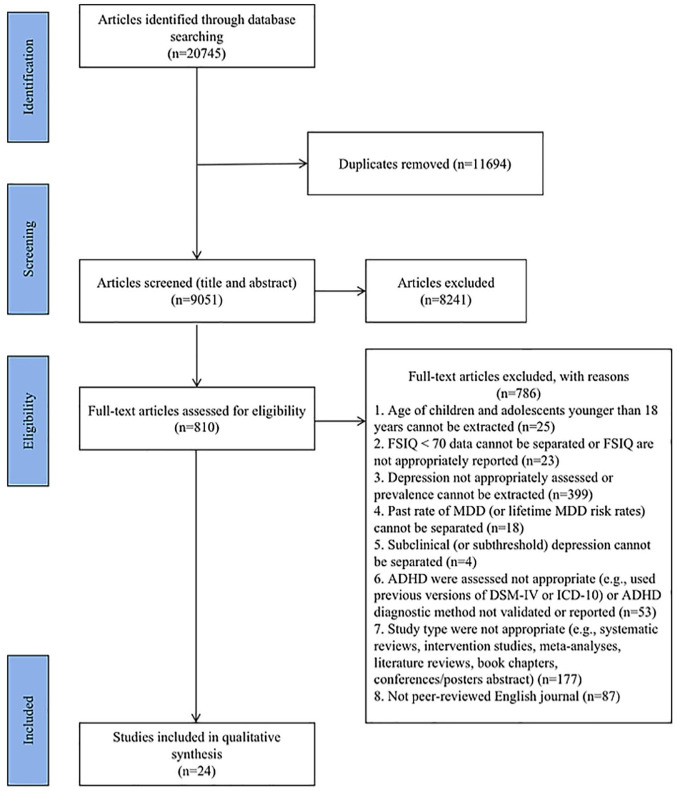
Flow chart of search strategy for this systematic review based on PRISMA.

The first author (S.W.) searched for articles in six databases based on the keywords in Table 1. According to the inclusion and exclusion criteria, S.W. independently screened the titles and abstracts of the retrieved articles. To ensure reliability, the second and third reviewers (I.O. and A.M.) independently screened 10% of the articles (5% each). The agreement rate at this stage was 96%. Following this, S.W. independently conducted a full-text screening of articles that passed the title and abstract screening stages according to the inclusion and exclusion criteria. The second and third reviewers (I.O. and A.M.) also independently screened 10% of these articles (5% each). The agreement rate was 92.5% at this stage. These agreement rates exceed the acceptable threshold of 80% reported in the literature (Belur et al., 2018). Differences at all stages of the screening process were resolved through discussion until a 100% consensus was reached. When final decisions remained unclear, two co-authors (T.S. and S.R.) were consulted for further clarification. To capture any relevant studies that might have been missed, a backward citation search was also conducted by reviewing the reference lists of studies included after the full-text screening; however, this search yielded no additional results. Figure 1 outlines the entire selection process.

### Data Extraction and Quality Assessment

Data were extracted from the included studies based on a customized data collection form from the Cochrane Handbook (Higgins & Thomas, 2023), the section related to intervention studies was removed from the original form and a new section on the rate of depression was added. Three reviewers extracted all data independently, with S.W. extracting data from all included studies and I.O. and A.M. each completing 5% of the data extraction (a total of 10%). The data extracted included: (1) Key study details (e.g., first author and publication year); (2) Study country; (3) Study type or design (e.g., cross-sectional and longitudinal); (4) Recruitment pathways (e.g., clinical and community); (5) Sample size of children and adolescents with ADHD (including participants’ sex distribution); (6) Participant age (mean, standard deviation [*SD*], and range); (7) Full-Scale IQ (FSIQ) of participants (mean, *SD*, and range, and the tool used for assessment); (8) Participant medication status; (9) Other demographic information (e.g., SES and ethnicity); (10) Name and type of standard used to diagnose ADHD; (11) Name and type of measure used to assess depressive symptoms; and (12) Rates of clinical cut-off range depression or MDD. Depression rates (expressed as a percentage) were calculated by dividing the number of participants with co-occurring ADHD and depression by the total ADHD sample size. For case-control studies, data on the neurotypical group, including sample size, sex distribution, and depression rates, were also extracted. Depression rates in neurotypical children and adolescents were similarly calculated by dividing the number of neurotypical participants with depression by their total sample sizes. There was 95% to 100% agreement across both extractions. Differences were resolved through discussion until a 100% consensus was reached. Information that was missing in the included studies was noted as not reported (NR).

This review evaluated the risk of bias—defined as a systematic error or deviation from the truth in results (Higgins & Thomas, 2023)—in included studies based on the validated risk of bias assessment instrument, the *Checklist for Prevalence Studies*, developed by Munn et al. (2014). This instrument was developed to evaluate the risk of bias in prevalence studies across a range of disorders and evaluate the internal and external validity of prevalence data from different study designs (Munn et al., 2014). The bias rating chart utilized in this systematic review (see Table 2) was adapted from quality assessment criteria employed in similar topic-focused systematic reviews that examined depression rates in children and adolescents with Autistic Spectrum Disorders (ASD) but without ID (Stewart et al., 2022; Wigham et al., 2017) and ADHD prevalence in children and adolescents with ASD but without ID (Eaton et al., 2023). The first author (S.W.) conducted the quality assessment of the included studies independently. To ensure consistency in this stage, 10% of the studies were also evaluated by I.O. and A.M. (each assessing 5%). There was 100% agreement. No studies were excluded due to the high risk of bias ratings.

**Table 2.** Bias Rating Chart.

Assessment item	Criteria	Risk of bias scores
Diagnosis of ADHD	A diagnosis of ADHD was made by a researcher using a checklist based on DSM-IV/ICD-10 criteria and their subsequent (or revised) versions; or a diagnosis made prior to the study, without method details.	0 (high risk)
	ADHD was diagnosed using a validated research tool (e.g., K-SADS) by a researcher trained in its administration.	1 (medium risk)
	A psychologist or psychiatrist provided ADHD clinical diagnosis following DSM-IV/ICD-10 criteria and their subsequent (or revised) versions.	2 (low risk)
Assessment of depression	Used non-standardized tools to assess depression.	0 (high risk)
	Depression was assessed using a subscale within a standardized generic instrument.	1 (medium risk)
	Depression was assessed using a depression-specific standardized assessment tool (e.g., questionnaire and semi-structured interview); or diagnosed by a psychiatrist/psychologist using DSM-IV/ICD-10 or subsequent versions.	2 (low risk)
Clear description of participants	Reporting of key demographic information is missing.	0 (high risk)
	Some demographic information was provided, or a source for obtaining further details was referenced.	1 (medium risk)
	Provided comprehensive demographic details, including mean age, age range, sex, ethnicity, socio-economic status, and ADHD medication use.	2 (low risk)
Clear description of the recruitment pool	No information was provided about the recruitment pool.	0 (high risk)
	Some details were provided about the recruitment pool.	1 (medium risk)
	A detailed description of the recruitment pool was given, covering the geographical location, referral method (e.g., self-referral and database), and recruitment setting (e.g., clinical and community).	2 (low risk)
The reliability and validity of depression measures in the ADHD population	No psychometric properties were reported for children and adolescents with ADHD.	0 (high risk)
	The measure shows some reliability and validity in children and adolescents with ADHD.	1 (medium risk)
	The measure is designed specifically for children and adolescents with ADHD, and has acceptable psychometric properties in this population.	2 (low risk)
Measures of FSIQ	No FSIQ information was provided.	0 (high risk)
	FSIQ of the participants was given (e.g., described as all participants had an FSIQ >70), but no specific measure was provided.	1 (medium risk)
	FSIQ measured during the study (e.g., using the WISC).	2 (low risk)

*Note.* Total score = X/12. 0–3 = high risk of bias; 4–8 = medium risk of bias; 9–12 = low risk of bias. DSM-IV = Diagnostic and Statistical Manual of Mental Disorders, Fourth Edition (American Psychiatric Association, 1994); ICD-10 = International Classification of Diseases, 10th Edition (World Health Organization, 1992); K-SADS = Schedule for Affective Disorders and Schizophrenia for School-Age Children (Kaufman et al., 1997); FSIQ = Full-scale intelligence quotient; WISC = the Wechsler Intelligence Scale for Children.

### Data Analysis

This systematic review combined meta-analysis and narrative synthesis to comprehensively examine depression rates in children and adolescents with ADHD. The meta-analysis quantified the pooled depression rate and explored factors contributing to heterogeneity across studies (Barendregt et al., 2013; Higgins & Thomas, 2023). Studies that could not be included in the meta-analysis, such as those lacking sufficient data for statistical analysis, reporting information in incompatible formats, or using non-comparable outcome measures, were synthesized narratively to ensure their findings contributed to the broader understanding of the topic.

The meta-analysis was conducted using R (version 4.4.2 with the *meta* package; Balduzzi et al., 2019; Schwarzer et al., 2015). A generic inverse-variance method with a random effect model was used (DerSimonian & Laird, 1986), as it accounts for variability between studies due to differences in populations, settings, assessment methods, and methodological quality. This approach also provides a high conservation null hypothesis (Han & Eskin, 2011; Higgins & Thomas, 2023). Freeman-Tukey double arcsine transformation of rate to stabilize the variance was used, which is a reliable and robust option to deal with the instability of variance of a proportion in the meta-analysis, especially for studies with extreme rate estimates (Doi & Xu, 2021; Freeman & Tukey, 1950). The pooled rate estimate with 95% confidence intervals (CI) was presented in a forest plot. Heterogeneity was assessed using Cochran’s *Q* test (χ^2^, statistic significant: *p* < .05) and *I*^2^ statistic (substantial heterogeneity: *I*^2^ > 50%; Higgins & Thomas, 2023 ). Subgroup analyses were conducted exclusively for the ADHD group to explore differences in depression rates across factors such as sex (male, female), recruitment setting (clinical, community), assessment tools (e.g., interviews, questionnaires), and informants (e.g., child, parent, child and parent), and risk of bias (low, medium, high). To compare depression rates between children and adolescents with ADHD and their neurotypical peers, where neurotypical peers were included in a study, a meta-analysis was conducted using data from case-control studies (*n* = 7) that reported rates for both groups.

**Figure 2.**
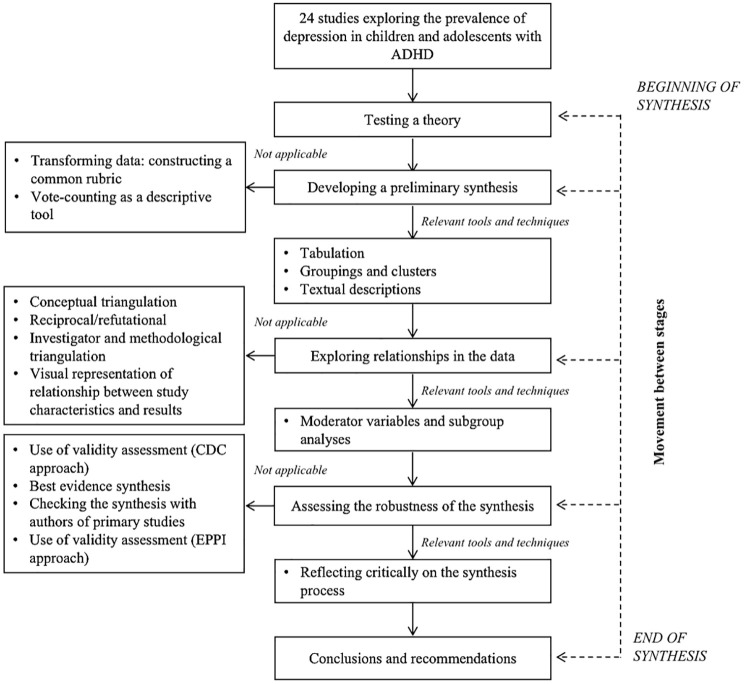
Systematic narrative synthesis framework. *Note*. CDC = Center for Disease Control; EPPI = Evidence for Policy and Practice Information and Co-ordinating Center.

The narrative synthesis was conducted following the guidance from the Economic and Social Research Council (ESRC) Methods Program, ensuring the results’ robustness and transparency. According to this narrative synthesis guidance, this systematic narrative synthesis was based on the framework of Figure 2 (Popay et al., 2006).

## Results

### Description of Included Studies

A total of 24 studies were included in this review, and their detailed characteristics are summarized in Table 3. Altogether, 6,815 participants were included in this review, consisting of 4,991 (73.24%) males and 1,824 (26.76%) females (excluding data from Sumiła & Cieślukowska, 2008, which did not provide subsample details—see Table 3). Among these participants, 5,047 were children and adolescents with ADHD, of whom 3,862 (76.52%) were male and 1,185 (23.48%) were female, with the distribution of male participants with ADHD ranging from 56.7% (Zerón-Rugerio et al., 2021) to 90.5% (Byun et al., 2006). ADHD sample sizes varied from 18 (Breaux et al., 2022) to 1000 (Inci et al., 2019), with participants’ ages spanning from 5 to 19 years old (Sumiła & Cieślukowska, 2008). Although this upper age limit exceeds the review’s criteria of 18 years or younger, Sumiła and Cieślukowska (2008) reported a mean sample age of 12.57 years, which is ≤18 years and therefore, they were retained. Three studies were also retained because they reported an average age of ≤18 years, despite not reporting a range of ages (Cleminshaw et al., 2020; Di Trani et al., 2014; S. Park et al., 2013).

**Table 3.** Demographic and Recruitment Pool Details, ADHD and Depression Assessment Methods, and Rates of Depression in the Studies Reviewed (*n* = 24).

Study	ADHD *n* (% male)	Age range (*M, SD*)	FSIQ range (*M, SD*), measure	SES	Ethnicity	ADHD medication	Recruitment pool (country)	ADHD assessment	Depression assessment	Rates of MDD (%)
Ahmed et al. (2021)	60 (68.3%)	6–11 years ADHD: *M* = 7.25–7.65, *SD* = 1.35–1.96 Neurotypical: *M* = 7.80, *SD* = 1.44	FSIQ > 70; *M* and *SD* NR; Wechsler Intelligence Scale.	NR	NR	Not receiving treatment	Clinical (Egypt): Child psychiatric outpatient clinic within the Department of Neurology and Psychiatry at Assiut University Hospital.	Diagnosed prior to the study by clinicians or professionals (criteria NR); Confirmed with parents: CPRS long format (Kao & Thomas, 2010).	Parent report: the CBCL depression sub-scale (Achenbach & Rescorla, 2001).	ADHD: 60% Neurotypical (*n* = 20; 50% males): 0%
Ambrosini et al. (2013)	500 (73.4%)	6.0–18.9 years (*M* = 10.2, *SD* = 3.2)	FSIQ > 75; *M, SD*, and measurement NR.	NR	Caucasian of European descent	NR	Clinical (United States): Local pediatric and behavioral health clinics for an ADHD genetic study.	Pre-study clinical ADHD diagnosis (name and type NR).	Interview: K-SADS-P-IVR, administered by a child psychiatrist trained in the reliability of K-SADS-P-IVR, respondents NR.	4.6%
Breaux et al. (2022)	18 (70%)	11–16 years (*M* = 13.5, *SD* = 1.6)	Range = 70–128; *M* = 100.56, *SD* = 17.12, WASI-II (Wechsler, 2011).	Mean household income ($) = 64,400 (*SD* = 37,700; range = 15,000–120,000).	55.6% black, 38.9% white, 5.6% bi- or multi-racial; 11.1% Latinx.	77.8% take ADHD medication (type and name NR).	Community (United States): flyers, posting on clinicaltrials.gov, emails to research participant databases and community clinic waitlists.	A comprehensive psychodiagnostics evaluation by clinicians (criteria NR); Confirmation: parent and child interview, using ChIPS (Weller et al., 2000; based on DSM-5); supplemented by parent and teacher report, using VADPRS and VADTRS (Wolraich et al., 1998, 2003).	Parent and child interview: ChIPS, administered by the principal investigator (first author).	44.40%
Byun et al. (2006)	105 (90.5%)	5–16 years (*M* = 9.70, *SD* = 2.48)	Excluded ID participants, *M* = 105.0, *SD* = 14.8; range and measurement NR.	NR	NR	NR	Clinical (South Korean): Outpatient and inpatient clinics at Samsung Medical Center.	Diagnosed prior to the study by clinicians or professionals (based on DSM-IV). Confirmation: parent and child interview, using the Korean version of K-SADS-PL (Kim et al., 2004), administered by six well-trained child and adolescent psychiatrists.	Parent and child interview: using the Korean version of K-SADS-PL, administered by well-trained child and adolescent psychiatrists.	10.50%
Cleminshaw et al. (2020)	174 (81.4%)	Range NR (*M* = 14.51, *SD* = 0.85)	FSIQ > 75; *M* = 95.51, *SD* = 13.04; WASI-II.	Household income: 36.7% less than $50,000; 55.9% $50,000 or above; 7.1% prefer not to answer	Race: Asian: 1.1%; black: 15.3%; white: 78.4%; other: 5.1%. Ethnicity: Hispanic or Latino: 10.2%; Not Hispanic or Latino: 88.8%	43% take ADHD medication (type and name NR).	Community (United States): High schools.	Parent report, using ARS-5 (DuPaul et al., 2016), based on DSM-5; Confirmation: parent interview, using the P-ChIPS (Weller et al., 1999), administered by trained research assistants	Children report: RADS-2 (Reynolds, 2004).	12%
Di Trani et al. (2014)	366 (87.7%)	Range NR (*M* = 105.15 months, *SD* = 30.26 months)	FSIQ > 70 (*M* = 98.12–101.56, *SD* = 8.92–14.91); WISC-III Italian version (Orsini & Picone, 2006).	*M* = 70.51, *SD* = 12.92, range NR, Index of Socioeconomic Status (Hollingshead, 1975).	Caucasian Italian	NR	Clinical (Italian): Regional center for ADHD in Rome.	Diagnosed prior to the study by an experienced child psychiatrist based on DSM-IV-TR. Confirmation: parent and teacher report, using the ARS-IV Italian version (Marzocchi & Cornoldi, 2000).	Parent and child interview: using the K-SADS-PL (Kaufman et al., 1997), administered by an experienced child psychiatrist.	4.37%
Elia et al. (2009)	500 (73.4%)	6–18 years (*M* = 10.2, *SD* = 3.2)	Range = 75–147, *M* = 104.4; median = 109; *SD* = 13.8; WASI.	M=42.2, *SD* = 10.7, median = 49, range NR, Index of Socioeconomic Status (Hollingshead, 1975).	European descent	NR	Clinical (United States): Pediatric and behavioral health clinics in Philadelphia.	Diagnosed prior to the study by clinicians (criteria NR). Confirmation: Parent and child interview, administered by a well-trained child psychiatrist, using the K-SADS-P-IVR.	Parent and child interview: assessed by K-SADS-P-IVR, administered by a child psychiatrist trained in the administration of K-SADS.	5.20%
S. S.-F. Gau, Lin, et al. (2010)	Baseline: 116 (sex distribution NR) Follow-up wave: 93 (82.8%; participation rate: 80.2%)	Baseline: Range NR (*M* = 7.3±2.8) Follow-up: 11–16 years ADHD: *M* = 13.2±1.5 Neurotypical: *M* = 13.3±1.4	FSIQ > 80; M, SD, and measurement NR.	Father’s education: 10.1% junior high school and below, 28.95% senior high school, 60.95% college and above; Maternal education: 9.05% junior high school and below, 39.65% senior high school, 51.3% college and above.	NR	86% have used ADHD medication; 82.8% used immediate-release methylphenidate.	Mixed (Taiwan): ADHD: Clinical; National Taiwan University Hospital. Neurotypical: Schools; same districts of ADHD group.	Diagnosed prior to the study by clinicians or professionals (DSM-IV). Confirmation: parent and child interview, using the Chinese K-SADS-E (S.-F. Gau & Suen Soong, 1999), administered by two well-trained interviewers majored in psychology and psychiatric nursing.	Parent and child interview: using the Chinese K-SADS-E, administered by well-trained interviewers majored in psychology and psychiatric nursing.	ADHD: 9.7% Neurotypical (*n* = 93; sex distribution NR): 3.2% (Baseline NR)
S. S.-F.Gau, Ni, et al. (2010)	296 (85.5%)	11–17 years; ADHD: *M* = 12.9; *SD* = 1.6 Neurotypical: *M* = 12.91; *SD* = 1.46	FSIQ ≥ 80; M and SD NR; WISC-III (Wechsler, 1991).	Father’s education: 39.8% senior high and below, 60.2% college and higher. Maternal education: 52.2% senior high and below, 47.8% college and higher.	NR	87.5% used methylphenidate; Mean duration (months)=19.57–20.28 (*SD* = 21.51–21.72)	Mixed (Taiwan): ADHD: Clinical; National Taiwan University Hospital. Neurotypical: Community; local schools.	Diagnosed prior to the study by clinicians, based on DSM-IV. Confirmation: Child and parent interview, using the Chinese K-SADS-E, administered by four well-trained interviewers.	Parent and child interview: using the Chinese K-SADS-E, administered by well-trained interviewers.	ADHD: 7.80% Neurotypical (*n* = 185; 72.4% males): 2.7%
Ghanizadeh (2008)	121 (71.1%).	5–16 years (*M* = 9.05, *SD* = 2.4).	FSIQ > 70, M, SD, and measurement NR.	Mean years of education: 9.9–10.8 (father); 9.9–10.4 (mother).	NR	NR	Clinical (Iran): The outpatient clinic at Hafez Hospital, affiliated with the Department of Child and Adolescent Psychiatry at Shiraz University of Medical Sciences.	Diagnosed prior to the study by clinicians based on DSM-IV-TR. Confirmation: parent and child interview, using the Farsi version of K-SADS (Ghanizadeh et al., 2006), administered by the trained child and adolescent psychiatrist.	Parent and child interview: using the Farsi version of K-SADS, administered by the trained child and adolescent psychiatrist.	2.5%
Hurtig et al. (2007)	105 (72.4%)	16–18 years (M and SD NR)	FSIQ > 70; M, and SD NR; WAIS-III (Wechsler, 1997).	Family income: 44.37% below average, 17.17% average, 38.47% above average.	NR	NR	Community (Finland): The study population was derived from the Northern Finland 1986 Birth Cohort.	Parents reported prior to the study, using the SWAN rating scale (J. Swanson et al., 2001). Confirmation: child and parent interview, using K-SADS-PL, administered by master-level interviewers.	Parent and child interview: using the K-SADS-PL, administered by the master-level interviewer.	ADHD: 9.50% Neurotypical (*n* = 172; 60% males): 5.2%
Inci et al. (2019)	1,000 (75.8%): ADHD-combined (ADHD-C): 571 (82.8%); ADHD-inattentive (ADHD-I): 379 (65.2%); ADHD-hyperactive/impulsive (ADHD-H): 30 (83.3%)	6–18 years (*M* = 10.82, *SD* = 3.24)	FSIQ > 70, M, SD, and measurement NR.	NR	NR	NR	Clinical (Turkey): Children hospitalized for psychiatric illness at Ege University.	Diagnosed prior to the study by psychiatrists using K-SADS-PL Turkish Version (Gökler et al., 2004). Confirmation: (1) Child interview, administered by psychiatrists using K-SADS-PL Turkish Version; (2) Parents and teachers report, using the T-DSM-IV-S (Ercan et al., 2001; Turgay, 1994).	Child interview: administered by psychiatrists using the K-SADS-PL Turkish Version.	7.2% ADHD-C: 8.1%; ADHD-I: 6.3%; ADHD-H: 3.3% Males: 6.2%; Females: 10.3%.
Ipci et al. (2020)	326 (73.9%)	8–15 years (*M* = 11.2, *SD* = 2.42)	FSIQ > 70, M, SD, and measurement NR.	Family income: 14.4% low-income, 46.3% middle-income, 39.3% high-income. Parental education: 3.3–5.9% elementary school, 12.6–14.1% middle school, 31.3–31.6% high school, 37.7–41.1% undergraduate degree, 7.7–15% postgraduate degree.	NR	NR	Clinical (Turkey): Izmir’s child and adolescent psychiatry clinics.	Diagnosed prior to the study by clinicians or professionals, based on DSM-IV-TR; supplemented with interview using the Turkish version of K-SADS-PL, administered by clinicians (respondents NR). Confirmation: Parents report, using the T-DSM-IV-S.	Interview: using the K-SADS-PL Turkish Version, administered by clinicians, respondents NR.	13.2%: ADHD-I: 12.8%; ADHD-H: 14.3%; ADHD-C: 10.5%. Males: 7.5%; Females: 29.4%.
Işık et al. (2018)	106 (79.25%)	8–16 years. (*M* = 10.1–10.2; *SD* = 1.8–2.1)	Excluded participants with ID (range NR). ADHD only: *M* = 96.9, *SD* = 14.8 ADHD + CD: *M* = 94.7, *SD* = 13.7. WISC-R Turkish version (Savaşır & Sahin, 1995).	NR	NR	Not receiving medication	Clinical (Turkey): Outpatient Clinic for Child and Adolescent Psychiatry at the Meram Faculty of Medicine, Necmettin Erbakan University.	Diagnosed prior to the study by clinicians based on DSM-5; supplemented with an interview using the K-SADS-PL Turkish Version, administered by child and adolescent psychiatrists. Confirmation: parent and teacher report, using the T-DSM-IV-S and the CTRS-RS (Kaner et al., 2013).	Child report: CDI Turkey version (Öy, 1991).	7.6%.
Joseph et al. (2019)	179 (69.3%)	6–8 years (*M* = 7.3, *SD* = 0.45)	FSIQ > 70; *M, SD*, and measurement NR.	Family annual income: 17% below $30,000, 26% $30,001–$60,000, 27% $60,001–$90,000, 28% above $90,001 Parents’ education: 69% completed high school, 46% completed university.	NR	14% Methylphenidate; 3% Clonidine; 1% Risperidone; 2% Melatonin; 1% Other.	Community (Australia): a community-based longitudinal study of children with and without ADHD (Children’s Attention Project).	Parents and teachers reported: using the 10-item Conners 3 ADHD Index (K. C. Conners, 2008). Confirmation: parent interview using the DISC-IV (Shaffer et al., 2000; based on DSM-IV-TR), administered by field staff who had a psychology degree.	Parent interview: using the DISC-IV, administered by field staff who had at least a 4-year undergraduate degree in psychology.	ADHD: 2%; Neurotypical (*n* = 212; 63.7% males): 0.5%
Mitchison and Njardvik (2019)	197 (73.6%)	8–15 years (*M* = 11.33, *SD* = 2.21)	Excluded participants with ID (*M, SD*, and range NR); WISC-IV (Wechsler, 2004).	NR	NR	NR	Clinical (Iceland): designated clinical institutions in Iceland.	Diagnosed prior to the study by clinicians or professionals (criteria NR). Parents and teachers report: ADHD Rating Scale. Confirmation: K-SADS-PL administered by clinicians (respondents NR).	Child report: using CDI (Kovacs, 1985).	21.43% Males: 18.75%; Females: 28.13%
Nadeau et al. (2015)	111 (64.9%)	8–17 years (*M* = 11.59, *SD* = 2.55)	FSIQ > 70; *M, SD*, and measurement NR.	Family income: 27% $40,000 or less, 37% $41,000–$82,000, 36% above $82,000.	90.1% Caucasian, 4.5% African American, 3.6% Hispanic, 0.9% Asian or other.	77.5% stimulant medication, 27% a serotonin reuptake inhibitor (SRI), 20.7% antihypertensive, 12.6% atypical antipsychotic, 11.7% non-SRI antidepressant	Clinical (United States): Outpatient pediatric psychiatric clinic specializing in the treatment of ADHD.	Diagnosed prior to the study by clinicians or professionals based on the DSM-IV-TR. Confirmation: Parent and child report, using the VADRS—P/C (Wolraich et al., 2003).	Child report: using the RCADS (Chorpita et al., 2000).	9%
Palacio-Ortiz et al. (2018)	228 (74.2%)	6–18 years (*M* and *SD* NR, Median age = 11.5)	FSIQ > 70, *M* and *SD* NR; WISC-IV.	22.8% high SES, 75.4% low SES.	NR	37.7% treated with prescription medications (type and name NR)	Clinical (Colombia): clinical setting in Medellin, Colombia.	An experienced child psychiatrist interviewed caregivers and children, based on the DSM-5.	Parent and child interview: administered by an experienced child psychiatrist, based on the DSM-5	15.4%
S. Park et al. (2013)	202 (86.1%)	Range NR (*M* = 9.0, *SD* = 2.5)	FSIQ > 71, *M* = 106, *SD* = 14.7, KEDI-WISC (K. S. Park et al., 1996).	NR	NR	NR	Clinical (South Korean): Child psychiatric clinics at university hospitals: Seoul National, Kyungpook National, and Chungbuk National Hospitals.	Diagnosed prior to the study by clinicians or professionals based on the DSM-IV-TR. Confirmation: parent report, using the ARS-IV Korean Version (So et al., 2002).	Child report: using the CDI Korean versions (Cho & Lee, 1990).	3.50%
Sumiła and Cieślukowska (2008)	62 (sex distribution NR).	5–19 years (*M* = 12.57, SD NR)	FSIQ > 70; *M, SD*, and measurement NR.	NR	NR	NR	Clinical (Poland): Psychological and Pedagogical Center.	Pre-study clinical ADHD diagnosis based on ICD-10, interviewed children/adolescents and their parents.	Child and parent interview: administered by psychiatrists using a structured clinical interview based on ICD-10.	27%
Suthar et al. (2018)	57 (71.9%)	6–12 years (M and SD NR).	FSIQ > 70; *M, SD*, and measurement NR.	5.3% upper SES, 31.6% upper middle SES, 38.6% lower middle SES, 12.3% upper lower SES, 12.3% lower SES.	NR	NR	Community (India): three different government primary schools in Bikaner city.	Parents and teachers reported: using the VADPRS and VADTRS (Wolraich et al., 1998, 2003). Confirmation: child interview, administered by the senior psychiatrist, according to DSM-IV-TR criteria.	Parent and teacher report: using the VADPRS and VADTRS. Supplemented with child interview: administered by the senior psychiatrist according to DSM-IV-TR criteria.	26.30%
Xia et al. (2015)	135 (73.3%)	7–10 years ADHD: *M* = 8.8, *SD* = 1.4; Neurotypical: *M* = 9.2, *SD* = 0.5	FSIQ > 70; *M, SD*, and measurement NR.	M (SD) years of fathers’ education: 14.9 (4.0) M (SD) years of mothers’ education: 12.4 (3.0) M (SD) annual household income in 10,000 Renminbi: 20.2 (9.3)	NR	NR	Clinical (Mainland China): Xin Hua Hospital pediatric department.	Parent report: the SNAP-IV (J. M. Swanson, 1992). Confirmation: child and parent interview, using the K-SADS-PL, administered by senior psychiatric clinicians.	Parent and child interview: using the K-SADS-PL, administered by senior psychiatrists. Supplemented with child report: the DSRSC (Birleson, 1981).	ADHD: 17.80%; Neurotypical (*n* = 65; 70.1% males): 0%
Yüce et al. (2013)	108 (76.9%). ADHD-C: 93 (82.8%) ADHD-I: 15 (40%)	6–18 years (*M* = 10.26, *SD* = 3.3)	FSIQ > 70; *M, SD*, and measurement NR.	NR	NR	NR	Clinical (Turkey): Department of Child and Adolescent Psychiatry, Faculty of Medicine, Ondokuz Mayıs Universit.	Diagnosed prior to the study by clinicians or professionals according to the DSM-IV criteria. Confirmation: child and parent interview, using K-SADS-PL Turkish version, administered by a trained clinician.	Parent and child interview: using the K-SADS-PL Turkish version, administered by trained physicians.	9.3. ADHD-C: 7.5%; ADHD-I: 20%. Male: 6%; female: 20%
Zerón-Rugerio et al. (2021)	60 (56.7%)	6–16 years; ADHD: *M* = 9.3, *SD* = 2.8; Neurotypical: *M* = 9.3, *SD* = 2.8	FSIQ > 70; *M, SD*, and measurement NR.	NR	NR	No pharmacological treatment	Mixed (Spain): ADHD (Clinical): The ADHD Unit within the Department of Child and Adolescent Psychiatry and Psychology at the Hospital of Sant Joan de Déu in Barcelona. Neurotypical (Mixed): ADHD group’s classmates (40%) or patients from other hospital services (60%).	Diagnosed prior to the study by an expert child psychiatrist, based on the DSM-IV-TR. Confirmation: Parents completed ARS-IV and the CPRS-R:S (C. K. Conners, 1997); Supplemented with interviews, using the K-SADS-PL Spanish Version (Ulloa et al., 2006; interviewers and respondents NR).	Interview: using the K-SADS-PL Spanish Version (interviewers and respondents NR).	ADHD: 1.70%; Neurotypical (*n* = 60; 56.7% males): 1.7%

*Note*. ID = intellectual disability; M = mean; SD = standard deviation; FSIQ = full-scale intelligence quotient; MDD = major depressive disorder; NR = not reported; ARS-IV = ADHD Rating Scale, Fourth Edition (DuPaul et al., 1998; Italian Version: Marzocchi & Cornoldi, 2000; Korean Version: So et al., 2002); ARS-5 = ADHD Rating Scale 5 Home Version (DuPaul et al., 2016); CBCL = Child Behavior Checklist (Achenbach & Rescorla, 2001); CDI = Children’s Depression Inventory (Kovacs, 1985; Turkey version: Öy, 1991; Korean version: Cho & Lee, 1990); ChIPS/P-ChIPS = Children’s Interview for Psychiatric Syndromes (Weller et al., 2000)/Children’s Interview for Psychiatric Syndromes-Parent Version (Weller et al., 1999); CPRS/CTRS-RS/CPRS-R:S = Conners Parent Rating Scale (Kao & Thomas, 2010)/Conners’ Teacher Rating Scale-Revised Short Version (C. K. Conners et al., 1998; Turkish Version: Kaner et al., 2013)/Conners’ Parent Rating Scale–Revised: Short Form (C. K. Conners, 1997); DISC-IV = Diagnostic Interview Schedule for Children, Version IV (Shaffer et al., 2000); DSM-IV/DSM-IV-TR/DSM-5 = Diagnostic and Statistical Manual of Mental Disorders, Fourth Edition (American Psychiatric Association, 1994)/Fourth Edition, Text Revision (American Psychiatric Association, 2000)/Fifth Edition (American Psychiatric Association, 2013); DSRSC = Depression Self-Rating Scale for Children (Birleson, 1981); ICD-10 = International Classification of Diseases, 10th Edition (World Health Organization, 1992); KEDI-WISC = Korean Educational Developmental Institute’s Wechsler Intelligence Scale for Children (K. S. Park et al., 1996); K-SADS = Schedule for Affective Disorders and Schizophrenia for School-Age Children (Kaufman et al., 1997; Farsi version: Ghanizadeh et al., 2006); Chinese K-SADS-E = Schedule for Affective Disorders and Schizophrenia for School-Age Children Epidemiologic version Chinese Version (S.-F. Gau & Suen Soong, 1999); K-SADS-P-IVR = Schedule for Affective Disorders and Schizophrenia for School-Age Children Present State version IV-Revised; K-SADS-PL = Schedule for Affective Disorders and Schizophrenia for School-Age Children Present and Lifetime version (Kaufman et al., 1997; Turkish version: Gökler et al., 2004; Korean Version: Kim et al., 2004; Spanish version: Ulloa et al., 2006); RADS-2 = Reynolds Adolescent Depression Scale, Second Edition (Reynolds, 2004); RCADS = Revised Child’s Anxiety and Depression Scale (Chorpita et al., 2000); SNAP-IV = Swanson, Nolan, and Pelham Teacher and Parent Rating Scale (J. M. Swanson, 1992); SWAN = Strengths and Weaknesses of ADHD Symptoms and Normal Behaviors (J. Swanson et al., 2001); T-DSM-IV-S = Turgay DSM-IV Disruptive Behavior Disorders Rating Scale—Teacher and Parent Form (Turgay, 1994); VADPRS/VADTRS = Vanderbilt ADHD Diagnostic Parent/Teacher Rating Scale (Wolraich et al., 1998, 2003); VADRS-P/C = Vanderbilt ADHD Diagnostic Rating Scale—Parent/Child (Wolraich et al., 2003);WAIS-III = The Wechsler Adult Intelligence Scale, Third Edition (Wechsler, 1997); WASI-II = Wechsler Abbreviated Scale of Intelligence Second Edition (Wechsler, 2011); WISC-III/IV/R = The Wechsler Intelligence Scale for Children, third Edition (Wechsler, 1991; Turkey version: Orsini & Picone, 2006)/fourth Edition /Revised Turkish Version (Savaşır & Sahin, 1995).

This review included a diverse range of study designs: cross-sectional designs (Ambrosini et al., 2013; Breaux et al., 2022; Byun et al., 2006; Cleminshaw et al., 2020; Di Trani et al., 2014; Elia et al., 2009; Ghanizadeh, 2008; Inci et al., 2019; Ipci et al., 2020; Işık et al., 2018; Mitchison & Njardvik, 2019; Nadeau et al., 2015; Sumiła & Cieślukowska, 2008; Suthar et al., 2018; Yüce et al., 2013), longitudinal designs (S. S.-F. Gau, Lin, et al., 2010), and case-control studies (Ahmed et al., 2021; S. S.-F. Gau, Ni, et al., 2010; Hurtig et al., 2007; Joseph et al., 2019; Palacio-Ortiz et al., 2018; S. Park et al., 2013; Xia et al., 2015; Zerón-Rugerio et al., 2021).

Six studies reported participants’ ethnicity (Ambrosini et al., 2013; Breaux et al., 2022; Cleminshaw et al., 2020; Di Trani et al., 2014; Elia et al., 2009; Nadeau et al., 2015). The ADHD medication use of participants was reported by 10 studies (Ahmed et al., 2021; Breaux et al., 2022; Cleminshaw et al., 2020; S. S.-F. Gau, Lin, et al., 2010; S. S.-F. Gau, Ni, et al., 2010; Işık et al., 2018; Joseph et al., 2019; Nadeau et al., 2015; Palacio-Ortiz et al., 2018; Zerón-Rugerio et al., 2021).

All studies reported participants’ FSIQ≥70, although 13 studies did not report the IQ measure used (Ambrosini et al., 2013; Byun et al., 2006; S. S.-F. Gau, Lin, et al., 2010; Ghanizadeh, 2008; Inci et al., 2019; Ipci et al., 2020; Joseph et al., 2019; Nadeau et al., 2015; Sumiła & Cieślukowska, 2008; Suthar et al., 2018; Yüce et al., 2013; Xia et al., 2015; Zerón-Rugerio et al., 2021). Two studies reported a higher threshold, with an FSIQ of 80 or above (S. S.-F. Gau, Lin, et al., 2010; S. S.-F. Gau, Ni, et al., 2010). Fourteen studies reported socioeconomic status (SES); eight provided data on parental income (Breaux et al., 2022; Cleminshaw et al., 2020; Di Trani et al., 2014; Elia et al., 2009; Hurtig et al., 2007; Nadeau et al., 2015; Palacio-Ortiz et al., 2018; Suthar et al., 2018), three reported parents’ education or occupation (S. S.-F. Gau, Lin, et al., 2010; S. S.-F. Gau, Ni, et al., 2010; Ghanizadeh, 2008), and three included both parents’ education or occupation and income (Ipci et al., 2020; Joseph et al., 2019; Xia et al., 2015).

In this review, included studies were conducted across various regions, with most published in Asia (*n* = 6), including three studies in China (including Taiwan; S. S.-F. Gau, Lin, et al., 2010; S. S.-F. Gau, Ni, et al., 2010; Xia et al., 2015), two in South Korea (Byun et al., 2006; S. Park et al., 2013), and one in India (Suthar et al., 2018). The Middle East had five studies, including four conducted in Turkey (Inci et al., 2019; Ipci et al., 2020; Işık et al., 2018; Yüce et al., 2013) and one in Iran (Ghanizadeh, 2008). North America was represented by five studies from the United States (Ambrosini et al., 2013; Breaux et al., 2022; Cleminshaw et al., 2020; Elia et al., 2009; Nadeau et al., 2015). Europe contributed five studies, with one each from Italy (Di Trani et al., 2014), Finland (Hurtig et al., 2007), Iceland (Mitchison & Njardvik, 2019), Poland (Sumiła & Cieślukowska, 2008), and Spain (Zerón-Rugerio et al., 2021). Other regions included Australia in Oceania (Joseph et al., 2019), Colombia in South America (Palacio-Ortiz et al., 2018), and Egypt in Africa (Ahmed et al., 2021), each represented by a single study.

### Measures of Depression

In this review, the included studies assessed depression in children and adolescents with ADHD using various methods (see Table 3), mainly interviews and questionnaires. Some included studies combined both methods: Suthar et al. (2018) employed senior psychiatrists to conduct interviews with children based on DSM-IV-TR, while depression ratings were also collected from parents and teachers using the Vanderbilt ADHD Diagnostic Parent/Teacher Rating Scale (VADPRS/VADTRS); Xia et al. (2015) involved senior psychiatrists interviewing children and their parents using the Kiddie Schedule for Affective Disorders and Schizophrenia for School-Age Children-Present and Lifetime Version (K-SADS-PL) while children self-reported depression scores via the Depression Self Rating Scale for Children (DSRSC).

Thirteen studies assessed depression through interviews, predominantly using the K-SADS and its different adaptations. The K-SADS is a semi-structured interview developed to evaluate present and past symptoms of affective and psychotic disorders (e.g., MDD, anxiety, ADHD, conduct disorder, and schizophrenia) in children and adolescents aged 6 to 18 years, and diagnoses are typically determined according to a combination of interview data obtained from the child and their parents (Kaufman et al., 1997). Among these 13 studies using the K-SADS, 9 explicitly reported interviewing both parents and children (Byun et al., 2006; Di Trani et al., 2014; Elia et al., 2009; S. S.-F. Gau, Lin, et al., 2010; S.S.-F. Gau, Ni, et al., 2010; Ghanizadeh, 2008; Hurtig et al., 2007; Xia et al., 2015; Yüce et al., 2013); one seemed to indicate that only children and adolescents were interviewed (i.e., “. . .The children and adolescents were first interviewed using the K-SADS-PL,” Inci et al., 2019, p. 1358); and three studies did not specify whether the interview was conducted with the child, the parent/guardian, or both (Ambrosini et al., 2013; Ipci et al., 2020; Zerón-Rugerio et al., 2021). For these three studies, this review assumes interviews included both the child and parent/guardian, which followed the interpretation of K-SADS in line with Kaufman et al. (1997), but caution should be given here. Of the 13 studies using the K-SADS, 10 employed interviews conducted by clinicians or psychiatrists (Ambrosini et al., 2013; Byun et al., 2006; Di Trani et al., 2014; Elia et al., 2009; Ghanizadeh, 2008; Inci et al., 2019; Ipci et al., 2020; Xia et al., 2015; Yüce et al., 2013; Zerón-Rugerio et al., 2021), while three used researcher or trained student research assistants (S. S.-F. Gau, Lin, et al., 2010; S. S.-F. Gau, Ni, et al., 2010; Hurtig et al., 2007). Two additional studies conducted interviews with both children/adolescents and parents, administered by psychiatrists and clinicians, but did not specify the tools used, only mentioning that they followed DSM-5 or ICD-10 criteria (Palacio-Ortiz et al., 2018; Sumiła & Cieślukowska, 2008). Another study conducted interviews with children, administered by senior psychiatrists, but only reported that these were based on DSM-IV-TR without specifying the tool used (Suthar et al., 2018). One study interviewed parents and children using the Children’s Interview for Psychiatric Syndromes (ChIPS), administered by researchers (Breaux et al., 2022). Another study interviewed parents using the Diagnostic Interview Schedule for Children, Version IV (DISC-IV), conducted by trained research assistants (Joseph et al., 2019).

Six studies used clinical cut-off scores from questionnaires to assess children and adolescents’ depression: one study reported by parents using the Child Behavior Checklist (CBCL; Ahmed et al., 2021); while the other five studies relied on child/adolescent self-report questionnaires, including the Reynolds Adolescent Depression Scale, Second Edition (RADS-2; Cleminshaw et al., 2020), the Children’s Depression Inventory (CDI; Işık et al., 2018; Mitchison & Njardvik, 2019; S. Park et al., 2013), and the Revised Child’s Anxiety and Depression Scale (RCADS; Nadeau et al., 2015).

### Measures of ADHD

The included studies utilized a range of approaches to assess ADHD (see Table 3), including prior diagnoses without further confirmation, prior diagnoses with confirmation in the study, ADHD diagnoses made in the study based on the DSM/ICD, and ADHD diagnoses in the study further validated by multiple sources.

Two studies relied solely on prior ADHD diagnoses; Ambrosini et al. (2013) did not report specific details, while Sumiła and Cieślukowska (2008) reported that ADHD was diagnosed based on ICD-10. Six studies confirmed prior clinical diagnoses using the K-SADS to interview both children and their parents (Byun et al., 2006; Elia et al., 2009; S. S.-F. Gau, Lin, et al., 2010; S. S.-F. Gau, Ni, et al., 2010; Ghanizadeh, 2008; Yüce et al., 2013). Four studies used parent- and/or teacher-reported questionnaires to confirm prior ADHD diagnoses: Ahmed et al. (2021) employed the Conners Parent Rating Scale (CPRS); Di Trani et al. (2014) used the ARS-IV reported by both parents and teachers; Nadeau et al. (2015) used the Vanderbilt ADHD Diagnostic Rating Scale-Parent/Child (VADRS-P/C) reported by children and parents; and S. Park et al. (2013) utilized the ARS-IV reported by parents. Five studies confirmed prior diagnoses with a combination of parent and/or teacher reported questionnaires (i.e., ARS, Conners’ Teacher Rating Scale-Revised Short [CTRS-RS], and T-DSM-IV-S) and interviews using the K-SADS (Inci et al., 2019; Ipci et al., 2020; Işık et al., 2018; Mitchison & Njardvik, 2019; Zerón-Rugerio et al., 2021).

One study, Palacio-Ortiz et al. (2018), diagnosed ADHD during the study using DSM-5 criteria by child psychiatrists. Six studies involved ADHD diagnoses made during the study using multiple sources: Breaux et al. (2022) diagnosed ADHD through clinicians and confirmed it using ChIPS interviews with parents alongside parent and teacher reports on the VADPRS/VADTRS; Cleminshaw et al. (2020) assessed ADHD with parent-reported ARS-5 scores, confirmed through ChIPS interviews with parents conducted by trained research assistants; Hurtig et al. (2007) collected parent-reported Strengths and Weakness of ADHD symptoms and Normal Behaviors (SWAN) ratings and confirmed ADHD through K-SADS-PL interviews with parents and children/adolescents administered by psychiatrists; Joseph et al. (2019) gathered ADHD symptoms using the 10-item Conner 3 ADHD Index from parents and teachers, confirmed through DISC-IV interviews with parents conducted by trained psychologists; Suthar et al. (2018) collected ADHD data via parent and teacher VADPRS/VADTRS reports and confirmed diagnoses using DSM-IV-TR-based interviews conducted by senior psychiatrists; and Xia et al. (2015) collected ADHD symptom ratings from parents using Swanson, Nolan, and Pelham Teacher and Parent Rating Scale (SNAP-IV) and a psychiatrist conducted K-SADS-PL interviews with children and parents to confirm ADHD.

### Quality Appraisal

Of these 24 included studies, 8 demonstrated a low overall risk of bias (Breaux et al., 2022; Cleminshaw et al., 2020; Işık et al., 2018; Mitchison & Njardvik, 2019; Nadeau et al., 2015; Palacio-Ortiz et al., 2018; S. Park et al., 2013; Suthar et al., 2018), while the remaining 16 had a medium overall risk of bias. None of the studies had a high overall risk of bias. Table 4 presents a detailed summary of the quality assessment results, including the risk of bias ratings for each item and the overall rating of the included studies.

**Table 4.** Results of the Quality Assessment.

Study	Diagnosis of ADHD	Assessment of depression	Clear description of participants	Clear description of the recruitment pool	The reliability and validity of depression measures in the ADHD population	Measures of FSIQ	Total risk
Ahmed et al. (2021)	Low risk	Medium risk	Medium risk	Low risk	High risk	Low risk	Medium risk
Ambrosini et al. 2013	High risk	Medium risk	Medium risk	Low risk	High risk	Medium risk	Medium risk
Breaux et al. (2022)	Low risk	Medium risk	Low risk	Low risk	High risk	Low risk	Low risk
Byun et al. (2006)	Low risk	Medium risk	Medium risk	Low risk	High risk	Medium risk	Medium risk
Cleminshaw et al. (2020)	Medium risk	Low risk	Low risk	Low risk	High risk	Low risk	Low risk
Di Trani et al. (2014)	Low risk	Medium risk	Medium risk	Medium risk	High risk	Low risk	Medium risk
Elia et al. (2009)	Low risk	Medium risk	Medium risk	Low risk	High risk	Low risk	Medium risk
S. S.-F. Gau, Lin, et al. (2010)	Low risk	Medium risk	Medium risk	Medium risk	High risk	Medium risk	Medium risk
S. S.-F. Gau, Ni, et al. (2010)	Low risk	Medium risk	Medium risk	Medium risk	High risk	Low risk	Medium risk
Ghanizadeh (2008)	Low risk	Medium risk	Medium risk	Low risk	High risk	Medium risk	Medium risk
Hurtig et al. (2007)	Medium risk	Medium risk	Medium risk	Medium risk	High risk	Low risk	Medium risk
Inci et al. (2019)	Low risk	Medium risk	Medium risk	Low risk	High risk	Medium risk	Medium risk
Ipci et al. (2020)	Low risk	Medium risk	Medium risk	Medium risk	High risk	Medium risk	Medium risk
Işık et al. (2018)	Low risk	Low risk	Medium risk	Low risk	High risk	Low risk	Low risk
Joseph et al. (2019)	Medium risk	Medium risk	Medium risk	Low risk	High risk	Medium risk	Medium risk
Mitchison and Njardvik (2019)	Low risk	Low risk	Medium risk	Low risk	High risk	Low risk	Low risk
Nadeau et al. (2015)	Low risk	Medium risk	Low risk	Low risk	Medium risk	Medium risk	Low risk
Palacio-Ortiz et al. (2018)	Low risk	Low risk	Medium risk	Low risk	Medium risk	Low risk	Low risk
S. Park et al. (2013)	Low risk	Low risk	Medium risk	Low risk	High risk	Low risk	Low risk
Sumiła and Cieślukowska (2008)	Low risk	Low risk	Medium risk	Medium risk	Medium risk	Medium risk	Medium risk
Suthar et al. (2018)	Low risk	Low risk	Medium risk	Low risk	Medium risk	Medium risk	Low risk
Xia et al. (2015)	Low risk	Low risk	Medium risk	Low risk	High risk	Medium risk	Medium risk
Yüce et al. (2013)	Low risk	Medium risk	Medium risk	Low risk	High risk	Medium risk	Medium risk
Zerón-Rugerio et al. (2021)	Low risk	Medium risk	Medium risk	Low risk	High risk	Medium risk	Medium risk

*Note*. FSIQ = full-scale IQ.

In terms of ADHD diagnosis, 20 were considered to have a low risk of bias. In these studies, ADHD was diagnosed by clinicians following DSM/ICD criteria, with further confirmation provided by a validated standardized research tool (e.g., ChIPS and K-SADS). Three studies were considered to be at medium risk in this item as they solely relied on validated tools (e.g., DISC-IV, K-SADS, and PChIPS) to diagnose ADHD without clinicians’ involvement (Cleminshaw et al., 2020; Hurtig et al., 2007; Joseph et al., 2019). One study was assessed as high risk as participants’ ADHD were diagnosed before the study but did not provide detailed information about the methodology (Ambrosini et al., 2013).

Regarding the assessment of depression, 16 studies were classified as having a medium risk due to the use of depression subscales from standardized universal measures; the remaining 8 were considered as low-risk as they used standardized assessment tools (or clinical interviews) specifically designed to assess depression, or a diagnosis made by clinicians or researchers based on DSM/ICD (Cleminshaw et al., 2020; Işık et al., 2018; Mitchison & Njardvik, 2019; Palacio-Ortiz et al., 2018; S. Park et al., 2013; Sumiła & Cieślukowska, 2008; Suthar et al., 2018; Xia et al., 2015).

Regarding the clear description of participants, three studies were considered to have a low risk of bias because they provided detailed information on key demographic characteristics, including mage age and age range, sex distribution, medication use, SES, and ethnicity (Breaux et al., 2022; Cleminshaw et al., 2020; Nadeau et al., 2015); the other 21 studies were assessed as medium risk due to incomplete or missing demographic information.

In the item “Clear description of the recruitment pool,” 18 studies adequately reported referral methods and study settings and were therefore rated low risk. Transparency in recruitment methods is an important aspect of methodological quality, as it enables researchers to better evaluate the potential generalizability of the findings (Munn et al., 2014). In contrast, the remaining six provided only partial details on these aspects and were thus rated medium risk of bias (Di Trani et al., 2014; S. S.-F. Gau, Lin, et al., 2010; S. S.-F. Gau, Ni, et al., 2010; Hurtig et al., 2007; Ipci et al., 2020; Sumiła & Cieślukowska, 2008).

Regarding the item “The reliability and validity of depression measures in the ADHD population,” 20 were assessed as having a high risk of bias as depression measures in these studies did not show any psychometric properties evidence in the ADHD population. Four were classified as medium risk of bias in this item: two studies used depression assessment questionnaires that showed acceptable reliability and validity in children and adolescents with ADHD (Nadeau et al., 2015; Suthar et al., 2018); the other two involved direct depression diagnoses by psychiatrists according to DSM-5/ICD-10 (Palacio-Ortiz et al., 2018; Sumiła & Cieślukowska, 2008).

Eleven of the 24 studies were considered to be at low risk in measures of full-scale IQ (FSIQ) because they provided FSIQ data and reported on the validated FSIQ assessment tools they used (Ahmed et al., 2021; Breaux et al., 2022; Cleminshaw et al., 2020; Di Trani et al., 2014; Elia et al., 2009; S. S.-F. Gau, Ni, et al., 2010; Hurtig et al., 2007; Işık et al., 2018; Mitchison & Njardvik, 2019; Palacio-Ortiz et al., 2018; S. Park et al., 2013). The other 13 studies were classified as having a medium risk of bias in this item because they did not report the specific tools used to measure FSIQ.

### Rates of Depression in ADHD

**Figure 3.**
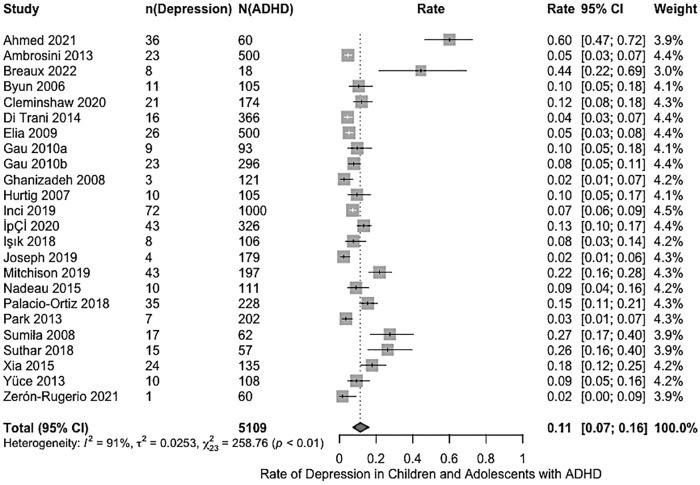
Forest plot of rates of depression of depression in children and adolescents with ADHD.

The reported rates of depression among children and adolescents with ADHD in the included studies varied widely, ranging from 1.7% to 60%. Using a random-effects model, the pooled estimated rate of depression was 11.31% (95% CI [0.07, 0.16]; *I*^2^ = 91%, τ^2^ = .03), as illustrated in the forest plot in Figure 3. To examine the robustness of this pooled estimate, a sensitivity analysis was conducted by using the leave-one-out approach, whereby excluding each study one-by-one from the analysis (Suvorov et al., 2023). The pooled rate remained stable, which varied between 10% (95% CI [0.07, 0.13]), with Ahmed et al. (2021) excluded, to 12% (95% CI [0.07, 0.17]), with Zerón-Rugerio et al. (2021) excluded. This indicated that no single study disproportionately influenced the pooled rate.

#### Rates by Measure Used to Assess Depression

The included studies employed various methods to assess depression in children and adolescents with ADHD, including interviews, questionnaires, or a combination of both. A subgroup analysis indicated significant differences in rates based on depression assessment methods (χ²(2) = 8.89, *p* < .05). Due to some depression measures having been used only in a single study (e.g., CBCL, ChIPS, RCADS, and DISC-IV), a subgroup analysis for specific measures was not conducted.

Studies employing mixed methods (combining questionnaires and interviews conducted by clinicians) reported the highest pooled rate of 20.87% (95% CI [0, 0.82]). Specifically, Suthar et al. (2018) employed VADPRS/VADTRS alongside DSM-IV-TR-based interview, reporting a rate of 26.30% (95% CI [0.16, 0.40]); and Xia et al. (2015) combined the DSRSC with the K-SADS-PL, reporting a rate of 17.8% (95% CI [0.12, 0.25]).

Studies using questionnaires reported a pooled depression rate of 16.13% (95% CI [0.02, 0.39]). Studies using versions of the CDI (*n* = 3) reported a total rate of 9.8% (95% CI [0, 0.41]; Işık et al., 2018; Mitchison & Njardvik, 2019; S. Park et al., 2013). Other tools included the CBCL, which reported a rate of 60% (95% CI [0.47, 0.72]; Ahmed et al., 2021); the RADS-2, with a rate of 12% (95% CI [0.23, 0.68]; Cleminshaw et al., 2020); and the RCADS, with a rate of 9% (95% CI [0.04, 0.16]; Nadeau et al., 2015).

Studies using interviews (*n* = 16) reported the lowest pooled rate (8.43%, 95% CI [0.05, 0.13]). Twelve studies using the K-SADS and its adaptations reported a total rate of 7.39% (95% CI [0.05, 0.10]; Ambrosini et al., 2013; Byun et al., 2006; Di Trani et al., 2014; Elia et al., 2009; S. S.-F. Gau, Lin, et al., 2010; S. S.-F. Gau, Ni, et al., 2010; Ghanizadeh, 2008; Hurtig et al., 2007; Inci et al., 2019; Ipci et al., 2020; Yüce et al., 2013; Zerón-Rugerio et al., 2021). Other interviews included the ChIPS (44.4%, 95% CI [0.23, 0.68]; Breaux et al., 2022); the DISC-IV (2%, 95% CI [0, 0.05]; Joseph et al., 2019); the DSM-5-based interview (15.4%, 95% CI [0.11, 0.21]; Palacio-Ortiz et al., 2018); and the ICD-10-based interview (27.4%, 95% CI [0.17, 0.40]; Sumiła & Cieślukowska, 2008). A subgroup analysis of these studies using interviews, categorized by administrators (clinicians vs. researchers), found no significant difference in depression rates (χ²(1) = 0.28, *p* = .60). The pooled rate was 8.01% (95% CI [0.04, 0.13]) for interviews conducted by clinicians/psychiatrists and 10.63% (95% CI [0.002, 0.31]) for those administered by trained researchers/research assistants.

#### Rates by Type of Informant

The included studies utilized various informants to assess depression in ADHD, including parent, child/adolescent, both parent and child/adolescent, and a combination of parent, child/adolescent, and teacher. Subgroup analysis indicated no significant differences in depression rates based on informant type (χ²(2) = 0.31, *p* = .86). The pooled depression rate was lowest in studies relying solely on children/adolescents as informants, at 9.52% (95% CI [0.04, 0.17]). Studies in which both parent and child/adolescent as informants reported a pooled rate of 9.73% (95% CI [0.06, 0.15]). The highest pooled rate of 24.37% (95% CI [0, 1]) was observed in studies using the parent as the sole informant. No comparison was conducted for the study incorporating teacher reports, as only one study (i.e., Suthar et al., 2018) met this criterion, reported a rate of 26.3% (95% CI [0.16, 0.39]).

#### Rates by Risk of Bias Rating

Subgroup analysis based on overall risk of bias ratings found no significant differences in depression rates between studies (χ²(1) = 1.06, *p* = 0.30). Studies with a medium overall risk of bias reported a pooled rate of depression of 9.85% (95% CI [0.05, 0.16]); while those with a low overall risk of bias had a slightly higher rate of 14.5% (95% CI [0.06, 0.25]).

Among other risk of bias items, most studies were rated as low or medium risk. However, the item assessing “the reliability and validity of depression measures specific to the ADHD population” showed a different rating pattern as no study was rated as low risk, and 20 studies were rated as high risk. To explore the potential impact of this imbalance, a subgroup analysis was conducted based on the rating of this item and indicated no significant differences between groups (χ²(1) = 2.75, *p* = .09). Studies rated as medium risk of this item had a higher pooled depression rate (18.22%, 95% CI [0.06, 0.35]) compared to those rated as high risk (10.1%, 95% CI [0.06, 0.16]).

#### Rates by Pubertal Status

According to the National Health Service (NHS), the typical onset of puberty occurs at around age 11 for girls and age 12 for boys (NHS, 2022). Based on this guidance, included studies were categorized into those focusing on estimated pre-pubertal children (mean age ≤12) and estimated post-pubertal adolescents (mean age >12). Subgroup analysis showed a higher estimated depression rate in the post-pubertal group (15.06%, 95% CI [0.05, 0.29]) compared to the pre-pubertal group (9.91%, 95% CI [0.05, 0.16]), although the difference was not significant (χ²(1) = 1.07, *p* = .30). Palacio-Ortiz et al. (2018) was not included in the subgroup analysis because they did not report the mean age (*SD*) of participants.

#### Rates by Sex

Four studies provided sex-specific depression rates (Inci et al., 2019; Ipci et al., 2020; Mitchison & Njardvik, 2019; Yüce et al., 2013). Subgroup analysis showed a significant difference in rate between sexes (χ²(1) = 5.09, *p* = .02), with females showing a higher rate (20.93%, 95% CI [0.07, 0.39]) than males (8.97%, 95% CI [0.02, 0.19]).

#### Rates by ADHD Medicine Use

Ten studies reported on ADHD medication use among participants (Ahmed et al., 2021; Breaux et al., 2022; Cleminshaw et al., 2020; S. S.-F. Gau, Lin, et al., 2010; S. S.-F. Gau, Ni, et al., 2010; Işık et al., 2018; Joseph et al., 2019; Nadeau et al., 2015; Palacio-Ortiz et al., 2018; Zerón-Rugerio et al., 2021). Subgroup analysis found no significant difference in depression rate between studies with and without participants receiving ADHD medication (χ²(1) = 0.17, *p* = .68). The pooled depression rate was 10.93% (95% CI [0.03, 0.22]) for studies reporting participants using ADHD medication and 17.78% (95% CI [0, 0.98]) for studies reporting participants not using ADHD medication.

#### Rates by Recruitment Pathways (Clinical or Community)

Subgroup analysis by recruitment settings showed no significant difference between groups (χ²(1) = 0.79, *p* = .50). Studies recruiting participants from community settings reported a pooled rate of 14.95% (95% CI [0.01, 0.38]), whereas those recruiting from clinical settings reported a lower pooled rate of 10.61% (95% CI [0.06, 0.16]).

### Comparison of Depression Rates Between ADHD and Neurotypical Participants

**Figure 4.**
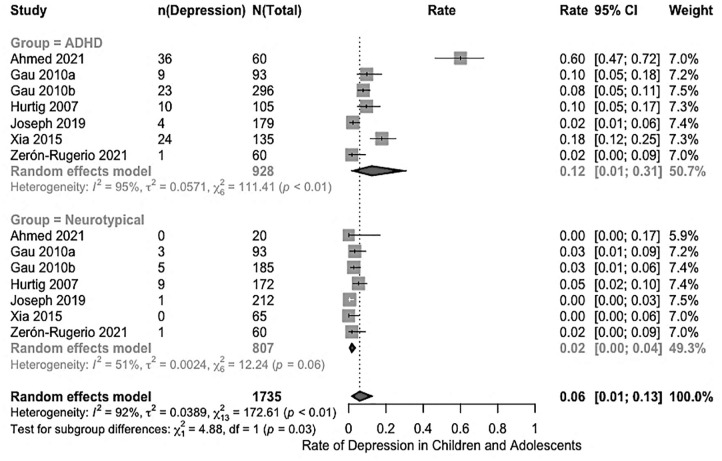
Forest plot comparing rates of depression in children and adolescents with ADHD to neurotypical children and adolescents in case-control studies.

A subgroup analysis of seven case-control studies compared depression rates between children and adolescents with ADHD and their neurotypical peers, where a neurotypical group was included in the current studies. The analysis indicated that the pooled depression rate was significantly higher in the ADHD group (12%, 95% CI [0.01, 0.31]) compared to the neurotypical group (2%, 95% CI [0.01, 0.04]; χ²(1) = 4.88, *p* = .03). Figure 4 presents a forest plot summarizing the pooled rate estimates and 95% CI for both groups.

## Discussion

This systematic review and meta-analysis examined depression rates in children and adolescents (≤18 years old) with ADHD but without ID, with reported rates varying from 1.7% to 60%. The meta-analysis estimated a pooled rate of 11.31% across 24 studies. The substantial heterogeneity (*I*^2^ = 91%) highlights the potential influence of various factors, including demographic characteristics (e.g., pubertal status, sex, and ADHD medication use), recruitment pathways (i.e., clinical and community), assessment tools (i.e., questionnaire and interviews), and informants (i.e., self and parent reports). Subgroup analyses were conducted to explore the sources of heterogeneity, indicating significant differences in depression rates in ADHD for sex and assessment tools, while no significant differences were found in others. Subgroup analyses for key factors such as SES, ethnicity, and geographical regions were not feasible due to inconsistent or absent reporting across studies. Nevertheless, depression frequently co-occurs in children and adolescents with ADHD, and rates in this population are significantly higher than for their neurotypical peers.

This review indicated sex and depression assessment tools as key factors significantly influencing the estimated rates of depression in children and adolescents with ADHD. Subgroup analysis suggested significant sex differences, with girls with ADHD having a higher pooled depression rate (20.93%) compared to boys (8.97%), approximately twice as high. A similar disparity is observed in their neurotypical peers, where depression rates in girls are two to three times higher than in boys (e.g., Frey et al., 2020; Salk et al., 2017). This pattern extends into adulthood; Hartman et al. (2023) demonstrating that while women with ADHD have higher depression rates than men with ADHD, the proportional sex differences remain consistent across those with and without ADHD. These findings suggest that ADHD may not alter the sex differences in depression rates. However, the underrepresentation of females in ADHD research may influence the accurate estimation of the depression rate in this population. This underrepresentation may stem from sex differences in ADHD symptoms, as females are more likely to show less hyperactivity/impulsive and disruptive behaviors, and more internalizing symptoms or eating disorders, which can lead to missed or delayed ADHD diagnosis in women and girls (Attoe & Climie, 2023; Quinn, 2008). Thus, some females with undiagnosed ADHD may be excluded from the ADHD group, leading to an underestimation of both the prevalence of ADHD and the associated rate of co-occurring depression. The predominance of male participants (over 70%) in most included studies highlights a longstanding sex imbalance in ADHD diagnosis. Further studies should strive for more sex-balanced samples, with a particular focus on females, given the recent increase in ADHD diagnoses among this group (Nussbaum, 2012; Young et al., 2020).

The methods used to assess depression also significantly influence rate estimates for children and adolescents with ADHD, with rates varying widely depending on the assessment method. The highest rates were reported in studies using both interviews and questionnaires (20.87%), followed by questionnaires alone (16.13%) and interviews alone (8.43%). This finding is consistent with previous research showing that prevalence estimates based on depression screening tools or rating scales tend to be higher than those based on interviews (Levis et al., 2019; Parsons et al., 2024). This review conducted a subgroup analysis that further indicated that depression rates reported through interviews were consistently low, regardless of whether clinicians (8%) or trained researchers (11%) administered the interviews. Compared to questionnaires or rating scales, interviews typically elicit more accuracy for diagnosing depression and estimating prevalence rates due to interviewer expertise and the supportive environment they provide for clarification (Phellas et al., 2012; Vassilopoulos et al., 2020). However, accurately assessing depression in children and adolescents with ADHD remains challenging due to the phenotypic overlap between the two conditions.

ADHD symptoms such as inattention, restlessness, and irritability also manifest in depressive symptomatology (Fraser et al., 2018; Powell et al., 2021), potentially leading children and their parents to overreport depressive symptoms on standardized questionnaires designed for neurotypical populations (Thapar et al., 2023). While interviews are generally more accurate for symptom evaluation, the lack of ADHD-specific validated measures may cause interviewers to interpret overlapping symptoms as part of ADHD, potentially masking and underestimating co-occurring depression (Garcia-Argibay et al., 2024). These contrasting biases highlight the importance of developing and/or adapting assessment tools tailored to ADHD populations. Furthermore, combining questionnaires and interviews may provide a more accurate assessment of depression, as questionnaires are effective for identifying specific depressive symptoms (e.g., low mood, concentration disturbance, sleep disturbances, and suicidal ideation), while interviews offer advantages in exploring contextual factors, clarifying symptoms, and evaluating their impact on functioning (Vassilopoulos et al., 2020).

In this review, only four employed tools were found to have acceptable psychometric properties for children and adolescents with ADHD. Nadeau et al. (2015) used the RCADS (Chorpita et al., 2000), a questionnaire that has demonstrated acceptable reliability, convergent validity, and discriminant validity in this population, particularly for the anxiety and depression subscales (Becker et al., 2019). Suthar et al. (2018) employed the VADPRS/VADTRS, a standardized questionnaire developed to assess ADHD and its co-occurring externalization (ODD/CD) and/or internalization (anxiety/depression) conditions in research and clinical settings (Wolraich et al., 1998, 2003). Additionally, two studies used diagnoses based on DSM-5 or ICD-10 interviews by experienced child psychiatrists (Palacio-Ortiz et al., 2018; Sumiła & Cieślukowska, 2008). These criteria, commonly used by psychiatrists, are the foundation of most depression measures and are considered acceptable for diagnosing depression in the ADHD population (Saito et al., 2010; Zimmerman et al., 2010). Most of the included studies used measures designed for the neurotypical population, given the overlapping symptoms of ADHD and depression, using specialized instruments designed or adapted for the ADHD population may help improve diagnostic accuracy (Thapar et al., 2023). The differences in reported depression rates between studies using measures with demonstrated reliability and validity for ADHD populations (18%) and those without such evidence (10%) suggest a need for further exploration of how assessment tools impact rate estimates. Further studies would benefit from exploring if developing or adapting existing measures to better account for the nuances of ADHD populations could enhance the accuracy of depression assessment. Future studies could also consider the combined use of questionnaires and interviews to provide a more comprehensive and accurate evaluation of depression in this population.

Other factors, including pubertal status, informant type, recruitment settings, and ADHD medication use, did not show statistically significant effects but may still influence depression rates in children and adolescents with ADHD. Puberty is a crucial developmental stage marked by physical changes, hormonal fluctuations, and social relationship challenges that can increase the risk of developing depression (Jiang et al., 2021; McGuire et al., 2019). This review found slightly higher depression rates in post-pubertal participants (15.1%) compared to pre-pubertal participants (9.9%), though the difference was not significant. The lack of significance may be due to the broad age ranges in some studies (e.g., 5–19 years) but classified them as pre-pubertal based solely on a mean age below 12 years (e.g., Ambrosini et al., 2013; Inci et al., 2019; Ipci et al., 2020; Nadeau et al., 2015; Zerón-Rugerio et al., 2021). Such classification may obscure developmental differences related to pubertal onset and introduce bias in depression rate estimates. Future research would benefit from focusing more selectively on different age stages or accurately assessing pubertal status to better understand the developmental trajectory of depression in this population.

This review found no significant differences in depression rates based on informants. However, trends indicated that studies only using parent reports had the highest pooled rates (24.37%), followed by combined parent and child reports (9.73%) and child/adolescent-only reports (9.52%). The lack of significance may be due to the limited number of studies exclusively using parent reports (*n* = 2), reducing the statistical power. These trends align with prior research suggesting that children and adolescents with ADHD tend to underestimate their depressive symptoms, while their parents may overreport them (Fraser et al., 2018). This underscores the importance of incorporating multiple informants in depression assessments to reduce bias in depression assessment (De Los Reyes & Kazdin, 2005; Tepper et al., 2008). Although teachers’ reports can offer valuable insights into depressive symptoms in social and academic contexts (Mesman & Koot, 2000; Thapar et al., 2023), only one study in this review included teachers as informants. Similarly, while clinicians’ observations provide a nuanced and multidimensional understanding of depression (Baik et al., 2008; Vares et al., 2015), none of the included studies used them as a primary source, suggesting a gap in the literature. Future research would benefit from integrating multiple informants—teachers, parents, children/adolescents, and clinicians—to enhance the validity and reliability of depression assessments in the ADHD population.

This review found no significant differences in depression rates based on recruitment settings. However, recruiting participants from both clinical and community settings remains important, as clinical samples often capture more severe ADHD and/or depressive symptoms due to stricter referral thresholds (Bauermeister et al., 2007; Orchard et al., 2017), while community samples reflect a broader range of symptom severities (e.g., subthreshold symptoms; Cuijpers et al., 2010; Seymour et al., 2014). Similarly, no significant differences were observed between studies with and without ADHD medication use. This may be attributed to inconsistencies or lack of the type of ADHD medication reported across included studies and variability in the proportion of participants receiving medication (ranging from 14% to 87.5%). Different medications, such as stimulants (e.g., methylphenidate) and non-stimulants (e.g., atomoxetine), can affect depressive symptoms through different mechanisms (Chang et al., 2016; Oh et al., 2022). Furthermore, a tendency for lower rates of ADHD medication use may also lead to under-treatment of co-occurring depression (Chang et al., 2016). These issues within the included studies likely introduced bias and reduced the reliability of the findings. To better understand the co-occurrence of ADHD and depression, future research would benefit from incorporating diverse recruitment sources and ensuring detailed reporting of ADHD medication use.

### Limitations and Future Directions

There are some limitations in this systematic review and meta-analysis. First, studies that used cut-off questionnaires to assess depression were included. While interviews may provide a more comprehensive evaluation of depressive symptoms (Craddock & Mynors-Wallis, 2014; Pettersson et al., 2018), questionnaires remain widely used in clinical practice, research, and screening. The Children’s Depression Inventory (CDI), for instance, is a well-researched and clinically valuable tool commonly applied to assess depression in pediatric psychiatric inpatients (Friedberg & Sinderman, 2011). Thus, including studies that employed these questionnaires was necessary for capturing the breadth of available research. Secondly, this review excluded studies not published in English, potentially limiting the inclusion of broader, regionally diverse studies.

This review was not able to examine the impact of SES, ethnicity, and geographical regions on depression rates in children and adolescents with ADHD due to inconsistent or absent reporting, and limited country representation in the included studies. Considering these factors is important, as clinical practices, diagnostic criteria, and cultural attitudes toward mental health can vary significantly across regions and populations (Krendl & Pescosolido, 2020; Rodríguez-Donate et al., 2024; Simon et al., 2002). Moreover, this review included some studies with small sample sizes (e.g., Breaux et al., 2022; sample size = 18). Small sample sizes are common in ADHD research due to the challenges of recruitment and the specificity of the population (Kanevski et al., 2023). However, studies with small sample sizes are more susceptible to variability and introduce “small-study effects,” where studies with small sample sizes tend to report larger prevalence rates or treatment effects due to factors such as publication bias, methodological differences, or greater sampling variability (Lin, 2018; Sterne et al., 2000). While these studies were retained due to their relevance to the aims of this review, the results should be interpreted with caution given the potential for bias.

It was beyond the scope of the current review to systematically examine factors and mechanisms contributing to differences in depression rates between children and adolescents with ADHD and their neurotypical peers. While a subgroup meta-analysis was conducted on the included case-control studies, moderate-to-high heterogeneity was observed in both groups, suggesting that methodological and sampling variability may have introduced bias. For example, most included studies (except Zerón-Rugerio et al., 2021) did not consistently report exclusion criteria for neurotypical participants, making it unclear whether these control groups recruited individuals who did not have any psychiatric conditions (i.e., “super-control” group; Tiego et al., 2023). Future studies would benefit from incorporating well-matched neurotypical control groups and applying consistent recruitment strategies (e.g., matching on age, recruitment settings, FSIQ, and co-occurring conditions apart from the ADHD diagnosis) to better examine the specificity of observed patterns.

Notwithstanding these limitations, this is the first systematic review and meta-analysis to identify depression rates in children and adolescents with ADHD. It underscores that depression is a common co-occurring condition in this population, with significantly higher rates than their neurotypical peers. This review highlights methodology diversity across studies in this field, including variations in recruitment pathways, depression assessment tools, and informants. It also points to the inconsistent reporting of crucial characteristics, including SES, ethnicity, pubertal status, and ADHD medication use, as well as the lack of safe and effective depression assessment tools tailored to children and adolescents with ADHD. To achieve more reliable and accurate rate estimates in further research, there should be a focus on recruiting large samples from community settings, using standardized depression assessment tools designed for the ADHD population, and collecting data from multiple informants, including children, parents, and teachers. This systematic review also highlights the need for further studies to explore if the developmental trajectory of depression in children and adolescents with ADHD follows patterns similar to those in neurotypical populations across different pubertal statuses and sex.

## References

[bibr1-10870547251341597] Achenbach T. M. Rescorla L. A. (2001). Manual for ASEBA school-age forms & profiles. University of Vermont, Research Center for Children, Youth & Families.

[bibr2-10870547251341597] Ahmed G. K. Darwish A. M. Khalifa H. Khashbah M. A. (2021). Evaluation of psychiatric comorbidity in attention-deficit hyperactivity disorder with epilepsy: A case-control study. Epilepsy Research, 169, 106505. 10.1016/j.eplepsyres.2020.106505 33302225

[bibr3-10870547251341597] Ahuja A. Martin J. Langley K. Thapar A. (2013). Intellectual disability in children with attention deficit hyperactivity disorder. Journal of Pediatrics, 163(3), 890–895.e1. 10.1016/j.jpeds.2013.02.043 PMC4078221 23608559

[bibr4-10870547251341597] Ambrosini P. J. Bennett D. S. Elia J. (2013). Attention deficit hyperactivity disorder characteristics: II. Clinical correlates of irritable mood. Journal of Affective Disorders, 145(1), 70–76. 10.1016/j.jad.2012.07.014 22868057 PMC3496809

[bibr5-10870547251341597] American Psychiatric Association. (1994). Diagnostic and statistical manual of mental disorders (4th ed.). American Psychiatric Publishing, Inc.

[bibr6-10870547251341597] American Psychiatric Association. (2000). Diagnostic and statistical manual of mental disorders, fourth edition, text revision (DSM-IV-TR) (4th ed., Vol. 1). 10.1176/appi.books.9780890423349

[bibr7-10870547251341597] American Psychiatric Association. (2013). Diagnostic and statistical manual of mental disorders (5th ed.). 10.1176/appi.books.9780890425596

[bibr8-10870547251341597] Antshel K. M. Zhang-James Y. Wagner K. E. Ledesma A. Faraone S. V. (2016). An update on the comorbidity of ADHD and ASD: A focus on clinical management. Expert Review of Neurotherapeutics, 16(3), 279–293. 10.1586/14737175.2016.1146591 26807870

[bibr9-10870547251341597] Ashwood K. L. Tye C. Azadi B. Cartwright S. Asherson P. Bolton P. (2015). Brief report: Adaptive functioning in children with ASD, ADHD and ASD + ADHD. Journal of Autism and Developmental Disorders, 45(7), 2235–2242. 10.1007/s10803-014-2352-y 25614019

[bibr10-10870547251341597] Attoe D. E. Climie E. A. (2023). Miss. Diagnosis: A systematic review of ADHD in adult women. Journal of Attention Disorders, 27(7), 645–657. 10.1177/10870547231161533 36995125 PMC10173330

[bibr11-10870547251341597] Babinski D. E. Waschbusch D. A. Waxmonsky J. G. (2019). Sex and pubertal status moderate the association between ADHD and depression symptoms. The Journal of Clinical Psychiatry, 80(3), 18m12548. 10.4088/JCP.18m12548 31120201

[bibr12-10870547251341597] Baik S.-Y. Bowers B. J. Oakley L. D. Susman J. L. (2008). What comprises clinical experience in recognizing depression? The primary care clinician’s perspective. The Journal of the American Board of Family Medicine, 21(3), 200–210. 10.3122/jabfm.2008.03.070258 18467531

[bibr13-10870547251341597] Balduzzi S. Rücker G. Schwarzer G. (2019). How to perform a meta-analysis with R: A practical tutorial. Evidence Based Mental Health, 22(4), 153–160. 10.1136/ebmental-2019-300117 31563865 PMC10231495

[bibr14-10870547251341597] Barendregt J. J. Doi S. A. Lee Y. Y. Norman R. E. Vos T. (2013). Meta-analysis of prevalence. Journal of Epidemiology and Community Health, 67(11), 974–978. 10.1136/jech-2013-203104 23963506

[bibr15-10870547251341597] Barkley R. A. (Ed.). (2015). Attention-deficit hyperactivity disorder: A handbook for diagnosis and treatment (4th ed.). The Guilford Press.

[bibr16-10870547251341597] Bauermeister J. J. Shrout P. E. Ramírez R. Bravo M. Alegría M. Martínez-Taboas A. Chávez L. Rubio-Stipec M. García P. Ribera J. C. Canino G. (2007). ADHD correlates, comorbidity, and impairment in community and treated samples of children and adolescents. Journal of Abnormal Child Psychology, 35(6), 883–898. 10.1007/s10802-007-9141-4 17505876 PMC3591788

[bibr17-10870547251341597] Becker S. Schindler D. Holdaway A. Tamm L. Epstein J. Luebbe A. (2019). The Revised Child Anxiety and Depression Scales (RCADS): Psychometric evaluation in children evaluated for ADHD. journal of psychopathology and behavioral assessment, 41(1), 93–106. 10.1007/s10862-018-9702-6 30930533 PMC6438181

[bibr18-10870547251341597] Belur J. Tompson L. Thornton A. Simon M. (2018). Interrater reliability in systematic review methodology: Exploring variation in coder decision-making. Sociological Methods & Research, 50(2), 837–865. 10.1177/0049124118799372

[bibr19-10870547251341597] Biederman J. Seidman L. J. Petty C. R. Fried R. Doyle A. E. Cohen D. R. Kenealy D. C. Faraone S. V. (2008). Effects of stimulant medication on neuropsychological functioning in young adults with attention-deficit/hyperactivity disorder. The Journal of Clinical Psychiatry, 69(7), 1150–1156. 10.4088/jcp.v69n0715 18517288

[bibr20-10870547251341597] Birleson P. (1981). The validity of depressive disorder in childhood and the development of a self-rating scale: A research report. Journal of Child Psychology and Psychiatry, 22(1), 73–88. 10.1111/j.1469-7610.1981.tb00533.x 7451588

[bibr21-10870547251341597] Breaux R. Eadeh H.-M. Swanson C. S. McQuade J. D. (2022). Adolescent emotionality and emotion regulation in the context of parent emotion socialization among adolescents with neurodevelopmental disorders: A call to action with pilot data. Research on Child and Adolescent Psychopathology, 50(1), 77–88. 10.1007/s10802-021-00833-w 34195911

[bibr22-10870547251341597] Byun H. Yang J. Lee M. Jang W. Yang J.-W. Kim J.-H. Hong S. D. Joung Y. S. (2006). Psychiatric comorbidity in Korean children and adolescents with attention-deficit hyperactivity disorder: Psychopathology according to subtype. Yonsei Medical Journal, 47(1), 113–113. 10.3349/ymj.2006.47.1.113 16502492 PMC2687567

[bibr23-10870547251341597] Chang Z. D’Onofrio B. M. Quinn P. D. Lichtenstein P. Larsson H. (2016). Medication for Attention-Deficit/Hyperactivity Disorder and risk for depression: A nationwide longitudinal cohort study. Biological Psychiatry, 80(12), 916–922. 10.1016/j.biopsych.2016.02.018 27086545 PMC4995143

[bibr24-10870547251341597] Cho S. Lee Y. (1990). Development of the Korean form of the Kovacs’ Children’s Depression Inventory. Journal of the Korean Neuropsychiatric Association, 29, 943–956.

[bibr25-10870547251341597] Choi W.-S. Woo Y. S. Wang S.-M. Lim H. K. Bahk W.-M. (2022). The prevalence of psychiatric comorbidities in adult ADHD compared with non-ADHD populations: A systematic literature review. PLoS ONE, 17(11), e0277175. 10.1371/journal.pone.0277175 PMC9635752 36331985

[bibr26-10870547251341597] Chorpita B. F. Yim L. Moffitt C. Umemoto L. A. Francis S. E. (2000). Assessment of symptoms of DSM-IV anxiety and depression in children: A revised child anxiety and depression scale. Behaviour Research and Therapy, 38(8), 835–855. 10.1016/S0005-7967(99)00130-8 10937431

[bibr27-10870547251341597] Cleminshaw C. L. DuPaul G. J. Kipperman K. L. Evans S. W. Owens J. S. (2020). Social deficits in high school students with attention-deficit/hyperactivity disorder and the role of emotion dysregulation. School Psychology, 35(4), 233–242. 10.1037/spq0000392 32673052

[bibr28-10870547251341597] Compas B. E. Oppedisano G. Connor J. K. Gerhardt C. A. Hinden B. R. Achenbach T. M. Hammen C. (1997). Gender differences in depressive symptoms in adolescence: Comparison of national samples of clinically referred and nonreferred youths. Journal of Consulting and Clinical Psychology, 65(4), 617–626. 10.1037/0022-006X.65.4.617 9256563

[bibr29-10870547251341597] Conners C. K. (1997). Conners’ rating scales–revised: Technical manual. Multi-Health Systems.

[bibr30-10870547251341597] Conners C. K. Sitarenios G. Parker J. D. A. Epstein J. N. (1998). The revised Conners’ Parent Rating Scale (CPRS-R): Factor structure, reliability, and criterion validity. Journal of Abnormal Child Psychology, 26(4), 257–268. 10.1023/A:1022602400621 9700518

[bibr31-10870547251341597] Conners K. C. (2008). Conners 3rd edition manual. Multi-Health Systems. Inc.

[bibr32-10870547251341597] Costello E. J. Copeland W. Angold A. (2011). Trends in psychopathology across the adolescent years: What changes when children become adolescents, and when adolescents become adults? Journal of Child Psychology and Psychi-atry, 52(10), 1015–1025. 10.1111/j.1469-7610.2011.02446.x PMC3204367 21815892

[bibr33-10870547251341597] Costello E. J. Erkanli A. Angold A. (2006). Is there an epidemic of child or adolescent depression? Journal of Child Psychology and Psychiatry, 47(12), 1263–1271. 10.1111/j.1469-7610.2006.01682.x 17176381

[bibr34-10870547251341597] Craddock N. Mynors-Wallis L. (2014). Psychiatric diagnosis: Impersonal, imperfect and important. British Journal of Psychiatry, 204(2), 93–95. 10.1192/bjp.bp.113.133090 24493652

[bibr35-10870547251341597] Cuffe S. Visser S. Holbrook J. Danielson M. Geryk L. Wolraich M. McKeown R. (2020). ADHD and psychiatric comorbidity: Functional outcomes in a school-based sample of children. Journal of Attention Disorders, 24(9), 1345–1354. 10.1177/1087054715613437 26610741 PMC4879105

[bibr36-10870547251341597] Cuijpers P. van Straten A. Warmerdam L. van Rooy M. J. (2010). Recruiting participants for interventions to prevent the onset of depressive disorders: Possible ways to increase participation rates. BMC Health Services Research, 10, 181. 10.1186/1472-6963-10-181 20579332 PMC2907376

[bibr37-10870547251341597] Davidsson M. Hult N. Gillberg C. Särneö C. Gillberg C. Billstedt E. (2017). Anxiety and depression in adolescents with ADHD and autism spectrum disorders; Correlation between parent- and self-reports and with attention and adaptive functioning. Nordic Journal of Psychiatry, 71(8), 614–620. 10.1080/08039488.2017.1367840 28836480

[bibr38-10870547251341597] Daviss W. (2008). A review of co-morbid depression in pediatric ADHD: Etiologies, phenomenology, and treatment. Journal of Child and Adolescent Psychopharmacology, 18(6), 565–571. 10.1089/cap.2008.032 19108661 PMC2699665

[bibr39-10870547251341597] De Los Reyes A. Kazdin A. E . (2005). Informant discrepancies in the assessment of childhood psychopathology: A critical review, theoretical framework, and recommendations for further study. Psychological Bulletin, 131(4), 483–509. 10.1037/0033-2909.131.4.483 16060799

[bibr40-10870547251341597] DerSimonian R. Laird N. (1986). Meta-analysis in clinical trials. Controlled Clinical Trials, 7(3), 177–188. 10.1016/0197-2456(86)90046-2 3802833

[bibr41-10870547251341597] Di Trani M. Di Roma F. Elda A. Daniela L. Pasquale P. Silvia M. Renato D . (2014). Comorbid depressive disorders in ADHD: The role of ADHD severity, subtypes and familial psychiatric disorders. Psychiatry Investigation, 11(2), 137. 10.4306/pi.2014.11.2.137 24843368 PMC4023087

[bibr42-10870547251341597] Doi S. A. Xu C. (2021). The Freeman–Tukey double arcsine transformation for the meta-analysis of proportions: Recent criticisms were seriously misleading. Journal of Evidence-Based Medicine, 14(4), 259–261. 10.1111/jebm.12445 34632718

[bibr43-10870547251341597] DuPaul G. J. Power T. J. Anastopoulos A. D. Reid R. (1998). ADHD Rating Scale—IV: Checklists, norms, and clinical interpretation. The Guilford Press.

[bibr44-10870547251341597] DuPaul G. J. Power T. J. Anastopoulos A. D. Reid R. (2016). ADHD Rating Scale-5 for children and adolescents: Checklists, norms, and clinical interpretation. The Guilford Press.

[bibr45-10870547251341597] Eaton C. Roarty K. Doval N. Shetty S. Goodall K. Rhodes S. M. (2023). The prevalence of Attention Deficit/Hyperactivity Disorder symptoms in children and adolescents with autism spectrum disorder without intellectual disability: A systematic review. Journal of Attention Disorders, 27(12), 1360–1376. 10.1177/10870547231177466 37287320 PMC10498659

[bibr46-10870547251341597] Elia J. Arcos-Burgos M. Bolton K. L. Ambrosini P. J. Berrettini W. Muenke M. (2009). ADHD latent class clusters: DSM-IV subtypes and comorbidity. Psychiatry Research, 170(2–3), 192–198. 10.1016/j.psychres.2008.10.008 19900717 PMC4131943

[bibr47-10870547251341597] Eng A. G. Phan J. M. Shirtcliff E. A. Eisenlohr-Moul T. A. Goh P. K. Martel M. M. (2023). Aging and pubertal development differentially predict symptoms of ADHD, depression, and impairment in children and adolescents: An eight-year longitudinal study. Research on Child and Adolescent Psychopathology, 51(6), 819–832. 10.1007/s10802-023-01030-7 36719623 PMC10198896

[bibr48-10870547251341597] Ercan E. S. Amado S. Somer O. (2001). Development of a test battery for the assessment of attention deficit hyperactivity disorder [in Turkish]. Çocuk ve Gençlik Ruh Sağlığı Dergisi, 8, 132–144.

[bibr49-10870547251341597] Fraser A. Cooper M. Agha S. S. Collishaw S. Rice F. Thapar A. Eyre O. (2018). The presentation of depression symptoms in attention-deficit/hyperactivity disorder: Comparing child and parent reports. Child and Adolescent Mental Health, 23(3), 243–250. 10.1111/camh.12253 30197576 PMC6120536

[bibr50-10870547251341597] Freeman M. F. Tukey J. W. (1950). Transformations related to the angular and the square root. The Annals of Mathematical Statistics, 21(4), 607–611.

[bibr51-10870547251341597] Frey M. Obermeier V. Von Kries R. Schulte-Körne G. (2020). Age and sex specific incidence for depression from early childhood to adolescence: A 13-year longitudinal analysis of German health insurance data. Journal of Psychiatric Research, 129, 17–23. 10.1016/j.jpsychires.2020.06.001 32554228

[bibr52-10870547251341597] Friedberg R. D. Sinderman S. A. (2011). CDI scores in pediatric psychiatric inpatients: A brief retrospective static group comparison. Depression Research and Treatment, 2011, Article 134179. 10.1155/2011/134179 PMC3130960 21747992

[bibr53-10870547251341597] Garcia-Argibay M. Brikell I. Thapar A. Lichtenstein P. Lundström S. Demontis D. Larsson H. (2024). Attention-Deficit/Hyperactivity Disorder and major depressive disorder: Evidence from multiple genetically informed designs. Biological Psychiatry, 95(5), 444–452. 10.1016/j.biopsych.2023.07.017 37562520

[bibr54-10870547251341597] Gau S.-F. Suen Soong W.-T. (1999). psychiatric comorbidity of adolescents with sleep terrors or sleepwalking: A case-control study. Australian & New Zealand Journal of Psychiatry, 33(5), 734–739. 10.1080/j.1440-1614.1999.00610.x 10544999

[bibr55-10870547251341597] Gau S. S.-F. Ni H.-C. Shang C.-Y. Soong W.-T. Wu Y.-Y. Lin L.-Y. Chiu Y.-N. (2010). Psychiatric comorbidity among children and adolescents with and without persistent Attention-Deficit Hyperactivity Disorder. Australian & New Zealand Journal of Psychiatry, 44(2), 135–143. 10.3109/00048670903282733 20113302

[bibr56-10870547251341597] Gau S. S.-F. Lin Y.-J. Tai-Ann Cheng A. Chiu Y.-N. Tsai W.-C. Soong W.-T. (2010). Psychopathology and symptom remission at adolescence among children with Attention-Deficit–Hyperactivity Disorder. Australian & New Zealand Journal of Psychiatry, 44(4), 323–332. 10.3109/00048670903487233 20307165

[bibr57-10870547251341597] Ghanizadeh A. (2008). ADHD, bruxism and psychiatric disorders: Does bruxism increase the chance of a comorbid psychiatric disorder in children with ADHD and their parents? Sleep and Breathing, 12(4), 375–380. 10.1007/s11325-008-0183-9 18421490

[bibr58-10870547251341597] Ghanizadeh A. Mohammadi M. R. Yazdanshenas A. (2006). Psychometric properties of the Farsi translation of the kiddie schedule for affective disorders and schizophrenia-present and lifetime version. BMC Psychiatry, 6(1), 10–10. 10.1186/1471-244X-6-10 16539703 PMC1484478

[bibr59-10870547251341597] Gökler B. Ünal F. Pehlivantürk B. Kültür E. Ç. Akdemir D. Taner Y. (2004). Okul Çaği Çocuklari İçin Duygulanim Bozukluklari ve Şizofreni Görüşme Çizelgesi -Şimdi ve Yaşam Boyu Şekli- Türkçe Uyarlamasinin Geçerlik ve Güvenirliği [Reliability and validity of schedule for affective disorders and schizophrenia for school age children—present and lifetime version—Turkish version (K-SADS-PL-T)]. Çocuk ve Gençlik Ruh Sağliği Dergisi, 11(3), 109–116.

[bibr60-10870547251341597] Goodman S. H. Lahey B. B. Fielding B. Dulcan M. Narrow W. Regier D. (1997). Representativeness of clinical samples of youths with mental disorders: A preliminary population-based study. Journal of Abnormal Psychology, 106(1), 3–14. 10.1037/0021-843X.106.1.3 9103713

[bibr61-10870547251341597] Han B. Eskin E. (2011). Random-effects model aimed at discovering associations in meta-analysis of genome-wide association studies. The American Journal of Human Genetics, 88(5), 586–598. 10.1016/j.ajhg.2011.04.014 21565292 PMC3146723

[bibr62-10870547251341597] Hankin B. L. Abela J. R. Z. (2005). Depression from childhood through adolescence and adulthood: A developmental vulnerability and stress perspective. In Hankin B. L. Abela J. R. (Eds.), Development of psychopathology: A vulnerability-stress perspective (pp. 245–288). SAGE Publications, Inc. 10.4135/9781452231655.n10

[bibr63-10870547251341597] Hankin B. L. Abramson L. Y. Moffitt T. E. Silva P. A. McGee R. Angell K. E. (1998). Development of depression from preadolescence to young adulthood: Emerging gender differences in a 10-year longitudinal study. Journal of Abnormal Psychology, 107(1), 128–140. 10.1037/0021-843X.107.1.128 9505045

[bibr64-10870547251341597] Hartman C. A. (2023). Epidemiology of ADHD coming of age and a plea for prospective research on causes and consequences of ADHD throughout the lifespan in multidisciplinary team science. JCPP Advances, 3(2), e12178. 10.1002/jcv2.12178 PMC10519730 37753162

[bibr65-10870547251341597] Hartman C. A. Larsson H. Vos M. Bellato A. Libutzki B. Solberg B. S. Chen Q. Du Rietz E. Mostert J. C. Kittel-Schneider S. Cormand B. Ribasés M. Klungsøyr K. Haavik J. Dalsgaard S. Cortese S. Faraone S. V. Reif A. (2023). Anxiety, mood, and substance use disorders in adult men and women with and without attention-deficit/hyperactivity disorder: A substantive and methodological overview. Neuroscience & Biobehavioral Reviews, 151, Article 105209. 10.1016/j.neubiorev.2023.105209 37149075

[bibr66-10870547251341597] Higgins J. Thomas J. (2023). Cochrane handbook for systematic reviews of interventions (2nd ed.). Wiley-Blackwell.

[bibr67-10870547251341597] Hollingshead A. A. (1975). Four-factor index of social status. Yale University.

[bibr68-10870547251341597] Hurtig T. Ebeling H. Taanila A. Miettunen J. Smalley S. McGough J. Loo S. Järvelin M.-R. Moilanen I. (2007). ADHD and comorbid disorders in relation to family environment and symptom severity. European Child & Adolescent Psychiatry, 16(6), 362–369. 10.1007/s00787-007-0607-2 17401612

[bibr69-10870547251341597] Inci S. B. Ipci M. Akyol Ardıç U. Ercan E. S. (2019). Psychiatric comorbidity and demographic characteristics of 1,000 children and adolescents with ADHD in Turkey. Journal of Attention Disorders, 23(11), 1356–1367. 10.1177/1087054716666954 27581245

[bibr70-10870547251341597] Ipci M. Izmir S. Turkcapar M. Ozdel K. Ardic U. Ercan E. (2020). Psychiatric comorbidity in the subtypes of ADHD in children and adolescents with ADHD according to DSM-IV. Noropsikiyatri Arsivi-Archives of Neuropsychiatry, 57(4), 283–289. 10.29399/npa.24807 PMC7735147 33354119

[bibr71-10870547251341597] Işık Ü. Bilgiç A. Toker A. Kılınç I . (2018). Serum levels of cortisol, dehydroepiandrosterone, and oxytocin in children with attention-deficit/hyperactivity disorder combined presentation with and without comorbid conduct disorder. Psychiatry Research, 261, 212–219. 10.1016/j.psychres.2017.12.076 29324397

[bibr72-10870547251341597] Jiang L. Yang D. Li Y. Yuan J. (2021). The influence of pubertal development on adolescent depression: The mediating effects of negative physical self and interpersonal stress. Frontiers in Psychiatry, 12, Article 786386. 10.3389/fpsyt.2021.786386 PMC8637052 34867564

[bibr73-10870547251341597] Johansson R. Carlbring P. Heedman Å. Paxling B. Andersson G. (2013). Depression, anxiety and their comorbidity in the Swedish general population: Point prevalence and the effect on health-related quality of life. PeerJ, 1, e98. 10.7717/peerj.98 PMC3709104 23862109

[bibr74-10870547251341597] Johnson D. Dupuis G. Piche J. Clayborne Z. Colman I. (2018). Adult mental health outcomes of adolescent depression: A systematic review. Depression and Anxiety, 35(8), 700–716. 10.1002/da.22777 29878410

[bibr75-10870547251341597] Joinson C. Heron J. Araya R. Paus T. Croudace T. Rubin C. Marcus M. Lewis G. (2012). Association between pubertal development and depressive symptoms in girls from a UK cohort. Psychological Medicine, 42(12), 2579–2589. 10.1017/S003329171200061X 22717026

[bibr76-10870547251341597] Joseph C. I. Evans S. Youssef G. J. Silk T. Anderson V. Efron D. Sciberras E. (2019). Characterisation of depressive symptoms in young children with and without attention deficit hyperactivity disorder. European Child & Adolescent Psychiatry, 28(9), 1183–1192. 10.1007/s00787-018-01274-5 30697638

[bibr77-10870547251341597] Kaner S. Büyüköztürk Ş ve İşeri E. (2013). Conners Anababa Dereceleme Ölçeği-Yenilenmiş Kısa: Türkiye stardardizasyon çalışması [Conners parent rating scale-revised short: Turkish standardisation study]. Noropsikiyatri Arşivi, 50(2), 100–109. https://www.dx.doi.org/10.4274/npa.y6219

[bibr78-10870547251341597] Kanevski M. Booth J. N. Stewart T. M. Rhodes S. M. (2023). Cognition and maths in children with Attention-Deficit/Hyperactivity disorder with and without co-occurring movement difficulties. Research in Developmental Disabilities, 136, Article 104471. 10.1016/j.ridd.2023.104471 36924616

[bibr79-10870547251341597] Kao G. S. Thomas H. M. (2010). Test review: C. Keith Conners Conners 3rd Edition Toronto, Ontario, Canada: Multi-health systems, 2008. Journal of Psychoeducational Assessment, 28(6), 598–602. 10.1177/0734282909360011

[bibr80-10870547251341597] Kaufman J. Birmaher B. Brent D. Rao U. Flynn C. Moreci P. Willamson D. Ryan N. (1997). Schedule for affective disorders and schizophrenia for school-age children-present and lifetime version (K-SADS-PL): Initial reliability and validity data. Journal of the American Academy of Child & Adolescent Psychiatry, 36(7), 980–988. 10.1097/00004583-199707000-00021 9204677

[bibr81-10870547251341597] Kessler R. C. Avenevoli S. Ries Merikangas K. (2001). Mood disorders in children and adolescents: An epidemiologic perspective. Biological Psychiatry, 49(12), 1002–1014. 10.1016/S0006-3223(01)01129-5 11430842

[bibr82-10870547251341597] Kim Y. S. Cheon K. A. Kim B. N. Chang S. A. Yoo H. J. Kim J. W. Cho S. C. Seo D. H. Bae M. O. So Y. K. Noh J. S. Koh Y. J. McBurnett K. Leventhal B. (2004). The reliability and validity of kiddie-schedule for affective disorders and schizophrenia-present and lifetime version- Korean version (K-SADS-PL-K). Yonsei Medical Journal, 45(1), 81–81. 10.3349/ymj.2004.45.1.81 15004873

[bibr83-10870547251341597] Kofler M. J. Spiegel J. A. Soto E. F. Irwin L. N. Wells E. L. Austin K. E. (2019). Do working memory deficits underlie reading problems in Attention-Deficit/Hyperactivity Disorder (ADHD)? Journal of Abnormal Child Psychology, 47(3), 433–446. 10.1007/s10802-018-0447-1 29923160 PMC6301149

[bibr84-10870547251341597] Kovacs M. (1985). The children’s depression, inventory (CDI). Psychopharmacology Bulletin, 21(4), 995–998. 4089116

[bibr85-10870547251341597] Krendl A. C. Pescosolido B. A. (2020). Countries and cultural differences in the stigma of mental illness: The East–West divide. Journal of Cross-Cultural Psychology, 51(2), 149–167. 10.1177/0022022119901297

[bibr86-10870547251341597] Lahey B. B. Hartung C. M. Loney J. Pelham W. E. Chronis A. M. Lee S. S. (2007). Are there sex differences in the predictive validity of DSM-IV ADHD among younger children? Journal of Clinical Child and Adolescent Psychology, 36(2), 113–126. 10.1080/15374410701274066 17484685

[bibr87-10870547251341597] Levis B. Yan X. W. He C. Sun Y. Benedetti A. Thombs B. D. (2019). Comparison of depression prevalence estimates in meta-analyses based on screening tools and rating scales versus diagnostic interviews: A meta-research review. BMC Medicine, 17(1), 65. 10.1186/s12916-019-1297-6 30894161 PMC6427845

[bibr88-10870547251341597] Lima N. N. R. do Nascimento V. B. de Carvalho S. M. F. de Abreu L. C. Neto M. L. R. Brasil A. Q. Junior F. T. C. de Oliveira G. F. Reis A. O. A. (2013). Childhood depression: A systematic review. Neuropsychiatric Disease and Treatment, 9, 1417–1425. 10.2147/NDT.S42402 24092979 PMC3788699

[bibr89-10870547251341597] Lin L. (2018). Bias caused by sampling error in meta-analysis with small sample sizes. PLoS ONE, 13(9), e0204056. 10.1371/journal.pone.0204056 PMC6136825 30212588

[bibr90-10870547251341597] Luby J. L. Essex M. J. Armstrong J. M. Klein M. H. Zahn-Waxler C. Sullivan J. P. Goldsmith H. H. (2009). Gender differences in emotional reactivity of depressed and at-risk preschoolers: Implications for gender specific manifestations of preschool depression. Journal of Clinical Child & Adolescent Psychology, 38(4), 525–537. 10.1080/15374410902976312 20183639 PMC2829727

[bibr91-10870547251341597] Lundervold A. J. Hinshaw S. P. Sorensen L. Posserud M.-B. (2016). Co-occurring symptoms of attention deficit hyperactivity disorder (ADHD) in a population-based sample of adolescents screened for depression. BMC Psychiatry, 16, 46. 10.1186/s12888-016-0739-3 26915733 PMC4768418

[bibr92-10870547251341597] Marzocchi G. Cornoldi C. (2000). An easy to use scale for the assessment of problematic behaviors in ADHD children. Psicol Clinic Sviluppo, 4, 43–64.

[bibr93-10870547251341597] Maughan B. Collishaw S. Stringaris A. (2013). Depression in childhood and adolescence. Journal of the Canadian Academy of Child and Adolescent Psychiatry, 22(1), 35–40. 23390431 PMC3565713

[bibr94-10870547251341597] McDougal E. Gracie H. Oldridge J. Stewart T. M. Booth J. N. Rhodes S. M. (2022). Relationships between cognition and literacy in children with attention-deficit/hyperactivity disorder: A systematic review and meta-analysis. British Journal of Developmental Psychology, 40(1), 130–150. 10.1111/bjdp.12395 34605577 PMC9292415

[bibr95-10870547251341597] McGuire T. C. McCormick K. C. Koch M. K. Mendle J. (2019). Pubertal maturation and trajectories of depression during early adolescence. Frontiers in Psychology, 10, Article 1362. 10.3389/fpsyg.2019.01362 PMC6582206 31244742

[bibr96-10870547251341597] McLeod G. F. H. Horwood L. J. Fergusson D. M. (2016). Adolescent depression, adult mental health and psychosocial outcomes at 30 and 35 years. Psychological Medicine, 46(7), 1401–1412. 10.1017/S0033291715002950 26818194

[bibr97-10870547251341597] Meinzer M. C. Chronis-Tuscano A. (2017). ADHD and the development of depression: Commentary on the prevalence, proposed mechanisms, and promising interventions. Current Developmental Disorders Reports, 4(1), 1–4. 10.1007/s40474-017-0106-1 33282629 PMC7717502

[bibr98-10870547251341597] Meinzer M. C. Pettit J. W. Viswesvaran C. (2014). The co-occurrence of attention-deficit/hyperactivity disorder and unipolar depression in children and adolescents: A meta-analytic review. Clinical Psychology Review, 34(8), 595–607. 10.1016/j.cpr.2014.10.002 25455624

[bibr99-10870547251341597] Mesman J. Koot H. M. (2000). Child-reported depression and anxiety in preadolescence: I. Associations with parent- and teacher-reported problems. Journal of the American Academy of Child & Adolescent Psychiatry, 39(11), 1371–1378. 10.1097/00004583-200011000-00011 11068892

[bibr100-10870547251341597] Mitchison G. M. Njardvik U. (2019). Prevalence and gender differences of ODD, anxiety, and depression in a sample of children with ADHD. Journal of Attention Disorders, 23(11), 1339–1345. 10.1177/1087054715608442 26443719

[bibr101-10870547251341597] Moher D. Liberati A. Tetzlaff J. Altman D. G. (2009). Preferred reporting items for systematic reviews and meta-analyses: The PRISMA statement. PLoS Medicine, 6(7), e1000097. 10.1371/journal.pmed.1000097 PMC2707599 19621072

[bibr102-10870547251341597] Munn Z. Moola S. Riitano D. Lisy K. (2014). The development of a critical appraisal tool for use in systematic reviews addressing questions of prevalence. International Journal of Health Policy and Management, 3(3), 123–128. 10.15171/ijhpm.2014.71 25197676 PMC4154549

[bibr103-10870547251341597] Nadeau J. M. Jacob M. L. Keene A. C. Alderman S. M. Hacker L. E. Cavitt M. A. Alvaro J. L. Storch E. A. (2015). Correlates and mediators of life satisfaction among youth with Attention-Deficit/Hyperactivity Disorder. Children’s Health Care, 44(2), 169–182. 10.1080/02739615.2014.896215

[bibr104-10870547251341597] NHS. (2022). Early or delay puberty. NHS. https://www.nhs.uk/conditions/early-or-delayed-puberty/

[bibr105-10870547251341597] Nussbaum N. L. (2012). ADHD and female specific concerns. Journal of Attention Disorders, 16(2), 87–100. 10.1177/1087054711416909 21976033

[bibr106-10870547251341597] Oh Y. Joung Y.-S. Kim J. (2022). Association between attention deficit hyperactivity disorder medication and depression: A 10-year follow-up self-controlled case study. Clinical Psychopharmacology and Neuroscience, 20(2), 320–329. 10.9758/cpn.2022.20.2.320 35466103 PMC9048009

[bibr107-10870547251341597] Orchard F. Pass L. Marshall T. Reynolds S. (2017). Clinical characteristics of adolescents referred for treatment of depressive disorders. Child and Adolescent Mental Health, 22(2), 61–68. 10.1111/camh.12178 32680323

[bibr108-10870547251341597] Orsini A. Picone L. (2006). WISC-III: Contributo Alla Taratura Italiana. Italian Version of Wechsler. Organizzazioni Speciali, Firenze.

[bibr109-10870547251341597] Öy B. (1991). Cocuklar icin depresyon olcegi: Gecerlik ve guvenirlik calismasi [Reliability and validity of children’s depression inventory]. Türk Psikiyatri Dergisi, 2, 132–136.

[bibr110-10870547251341597] Palacio-Ortiz J. D. Gomez-Cano S. Aguirre-Acevedo D. C. (2018). Sleep problems and profiles in attention deficit hyperactivity disorder assessed by the Children Sleep Habits Questionnaire-Abbreviated in Colombia. Salud Mental, 41(6), 261–269. 10.17711/SM.0185-3325.2018.038

[bibr111-10870547251341597] Park K. S. Yoon J. Y. Park H. J. Park H. J. Kwon K. U. (1996). Development of KEDI-WISC, individual intelligence test for Korean children. Korean Educational Development Institute.

[bibr112-10870547251341597] Park S. Jung S.-W. Kim B.-N. Cho S.-C. Shin M.-S. Kim J.-W. Yoo H. J. Cho D.-Y. Chung U.-S. Son J.-W. Kim H.-W. (2013). Association between the GRM7 rs3792452 polymorphism and attention deficit hyperacitiveity disorder in a Korean sample. Behavioral and Brain Functions, 9(1), 1. 10.1186/1744-9081-9-1 PMC3680053 23295062

[bibr113-10870547251341597] Parsons M. Qiu L. Levis B. Fan S. Sun Y. Amiri L. S. N. Harel D. Markham S. Vigod S. N. Ziegelstein R. C. Wu Y. Boruff J. T. Cuijpers Gilbody S. Patten S. B. Benedetti A. Thombs B. D. , & the DEPRESsion Screening Data (DEPRESSD) GDS Group. (2024). Depression prevalence of the Geriatric Depression Scale-15 was compared to Structured Clinical Interview for DSM using individual participant data meta-analysis. Scientific Reports, 14, Article 17430. 10.1038/s41598-024-68496-3 PMC11286862 39075146

[bibr114-10870547251341597] Patel V. Flisher A. J. Hetrick S. McGorry P. (2007). Mental health of young people: A global public-health challenge. The Lancet, 369(9569), 1302–1313. 10.1016/S0140-6736(07)60368-7 17434406

[bibr115-10870547251341597] Pearson D. A. Lachar D. Loveland K. A. Santos C. W. Faria L. P. Azzam P. N. Hentges B. A. Cleveland L. A. (2000). Patterns of behavioral adjustment and maladjustment in mental retardation: Comparison of children with and without ADHD. American Journal on Mental Retardation, 105(4), 236. 10.1352/0895-8017(2000)105<0236:POBAAM>2.0.CO;2 10934566

[bibr116-10870547251341597] Pettersson A. Modin S. Wahlström R. af Winklerfelt Hammarberg S. Krakau I. (2018). The Mini-Inter-national Neuropsychiatric Interview is useful and well accepted as part of the clinical assessment for depression and anxiety in primary care: A mixed-methods study. BMC Family Practice, 19(1), 19. 10.1186/s12875-017-0674-5 29368585 PMC5781342

[bibr117-10870547251341597] Phellas C. N. Bloch A. Seale C. (2012). Structured methods: Interviews, questionnaires and observation. In Seale C. (Ed.), Researching society and culture (3rd ed., pp. 182–202). SAGE Publications, Inc.

[bibr118-10870547251341597] Polanczyk G. V. Willcutt E. G. Salum G. A. Kieling C. Rohde L. A. (2014). ADHD prevalence estimates across three decades: An updated systematic review and meta-regression analysis. International Journal of Epidemiology, 43(2), 434–442. 10.1093/ije/dyt261 24464188 PMC4817588

[bibr119-10870547251341597] Popay J. Roberts H. Sowden A. Petticrew M. Arai L. Rodgers M. Britten N. Roen K. Duffy S. (2006). Guidance on the conduct of narrative synthesis in systematic reviews. A Product from the ESRC Methods Programme, 1(1), b92. 10.13140/2.1.1018.4643

[bibr120-10870547251341597] Powell V. Martin J. Thapar A. Rice F. Anney R. J. L. (2021). Investigating regions of shared genetic variation in attention deficit/hyperactivity disorder and major depressive disorder: A GWAS meta-analysis. Scientific Reports, 11(1), 7353. 10.1038/s41598-021-86802-1 33795730 PMC8016853

[bibr121-10870547251341597] Quinn P. O. (2008). Attention-deficit/hyperactivity disorder and its comorbidities in women and girls: An evolving picture. Current Psychiatry Reports, 10(5), 419–423. 10.1007/s11920-008-0067-5 18803916

[bibr122-10870547251341597] Reynolds W. M. (2004). The Reynolds Adolescent Depression Scale-Second Edition (RADS-2). In Hilsenroth M. J. Segal D. L. (Eds.), Comprehensive handbook of psychological assessment (Vol. 2, pp. 224–236). John Wiley & Sons, Inc.

[bibr123-10870547251341597] Rhodes S. M. Park J. Seth S. Coghill D. R. (2012). A comprehensive investigation of memory impairment in attention deficit hyperactivity disorder and oppositional defiant disorder. Journal of Child Psychology and Psychiatry, 53(2), 128–137. 10.1111/j.1469-7610.2011.02436.x 21770938

[bibr124-10870547251341597] Rodríguez-Donate M. C. Nieto-González I. L. Guirao-Pérez G. (2024). Country differences in the effects of individual traits on depression in women in Europe. International Journal of Mental Health and Addiction. Advance online publication. 10.1007/s11469-024-01289-x

[bibr125-10870547251341597] Rucklidge J. J. (2008). Gender differences in ADHD: Implications for psychosocial treatments. Expert Review of Neurotherapeutics, 8(4), 643–655. 10.1586/14737175.8.4.643 18416665

[bibr126-10870547251341597] Saito M. Iwata N. Kawakami N. Matsuyama Y. Ono Y. Nakane Y. Nakamura Y. Tachimori H. Uda H. Nakane H. Watanabe M. Naganuma Y. Furukawa T. A. Hata Y. Kobayashi M. Miyake Y. Takeshima T. Kikkawa T. (2010). Evaluation of the DSM-IV and ICD-10 criteria for depressive disorders in a community population in Japan using item response theory. International Journal of Methods in Psychiatric Research, 19(4), 211–222. 10.1002/mpr.320 20645305 PMC3671887

[bibr127-10870547251341597] Salk R. H. Hyde J. S. Abramson L. Y. (2017). Gender differences in depression in representative national samples: Meta-analyses of diagnoses and symptoms. Psychological Bulletin, 143(8), 783–822. 10.1037/bul0000102 28447828 PMC5532074

[bibr128-10870547251341597] Savaşır I. Sahin N. (1995). Wechsler Çocuklar Ýçin Zeka Ölçeði (WISC-R). Türk Psikologlar Derneði.

[bibr129-10870547251341597] Sayers A. (2008). Tips and tricks in performing a systematic review. British Journal of General Practice, 58(547), 136–136. 10.3399/bjgp08X277168 PMC2233974 18307870

[bibr130-10870547251341597] Schwarzer G. Carpenter J. R. Rücker G. (2015). Meta-analysis with R. Springer International Publishing. 10.1007/978-3-319-21416-0

[bibr131-10870547251341597] Seymour K. E. Chronis-Tuscano A. Iwamoto D. K. Kurdziel G. MacPherson L. (2014). Emotion regulation mediates the association between ADHD and depressive symptoms in a community sample of youth. Journal of Abnormal Child Psychology, 42, 611–621. 10.1007/s10802-013-9799-8 24221724 PMC4207628

[bibr132-10870547251341597] Shaffer D. Fisher P. Lucas C. P. Dulcan M. K. Schwab-Srone M. E. (2000). NIMH diagnostic interview schedule for children version IV (NIMH DISC-IV): Description, differences from previous versions, and reliability of some common diagnoses. Journal of the American Academy of Child & Adolescent Psychiatry, 39(1), 28–38. 10.1097/00004583-200001000-00014 10638065

[bibr133-10870547251341597] Shorey S. Ng E. D. Wong C. H. J. (2022). Global prevalence of depression and elevated depressive symptoms among adolescents: A systematic review and meta-analysis. British Journal of Clinical Psychology, 61(2), 287–305. 10.1111/bjc.12333 34569066

[bibr134-10870547251341597] Simon G. E. Goldberg D. P. Von Korff M. Üstun T. B. (2002). Understanding cross-national differences in depression prevalence. Psychological Medicine, 32(4), 585–594. 10.1017/S0033291702005457 12102373

[bibr135-10870547251341597] So Y. K. Noh J. S. Kim Y. S. Ko S. G. Koh Y. J. (2002). The reliability and validity of Korean parent and teacher ADHD rating scale. Journal of Korean Neuropsychiatric Association, 41(2), 283–289.

[bibr136-10870547251341597] Solberg B. S. Halmøy A. Engeland A. Igland J. Haavik J. Klungsøyr K. (2018). Gender differences in psychiatric comorbidity: A population-based study of 40 000 adults with attention deficit hyperactivity disorder. Acta Psychiatrica Scandinavica, 137(3), 176–186. 10.1111/acps.12845 29266167 PMC5838558

[bibr137-10870547251341597] Sterne J. A. C. Gavaghan D. Egger M. (2000). Publication and related bias in meta-analysis. Journal of Clinical Epidemiology, 53(11), 1119–1129. 10.1016/S0895-4356(00)00242-0 11106885

[bibr138-10870547251341597] Stewart T. M. Martin K. Fazi M. Oldridge J. Piper A. Rhodes S. M. (2022). A systematic review of the rates of depression in autistic children and adolescents without intellectual disability. Psychology and Psychotherapy: Theory, Research and Practice, 95(1), 313–344. 10.1111/papt.12366 34605156

[bibr139-10870547251341597] Stumper A. Alloy L. B. (2023). Associations between pubertal stage and depression: A systematic review of the literature. Child Psychiatry & Human Development, 54(2), 312–339. 10.1007/s10578-021-01244-0 34529199 PMC11869324

[bibr140-10870547251341597] Sumiła A. Cieślukowska A. M. (2008). Depressive symptomatology in young patients with ADHD in outpatient care. Acta Neuropsychologica, 6(4), 380–389.

[bibr141-10870547251341597] Suthar N. Garg N. Verma K. K. Singhal A. Singh H. Baniya G. (2018). Prevalence of Attention-deficit hyperactivity disorder in primary school children: A cross-sectional study. Journal of Indian Association for Child and Adolescent Mental Health, 14(4), 74–88.

[bibr142-10870547251341597] Suvorov A. Yu. Latushkina I. V. Gulyaeva K. А. Bulanov N. M. Nadinskaia M. Yu. Zaikin A. A. (2023). Basic aspects of meta-analysis. Part 1. Sechenov Medical Journal, 14(1), 4–14. 10.47093/2218-7332.2023.14.1.4-14

[bibr143-10870547251341597] Swanson J. Deutsch C. Cantwell D. Posner M. Kennedy J. L. Barr C. L. Moyzis R. Schuck S. Flodman P. Spence M. A. Wasdell M. (2001). Genes and attention-deficit hyperactivity disorder. Clinical Neuroscience Research, 1(3), 207–216. 10.1016/S1566-2772(01)00007-X

[bibr144-10870547251341597] Swanson J. M. (1992). School-based assessments and interventions for ADD students. KC publishing.

[bibr145-10870547251341597] Tepper P. Liu X. Guo C. Zhai J. Liu T. Li C. (2008). Depressive symptoms in Chinese children and adolescents: Parent, teacher, and self reports. Journal of Affective Disorders, 111(2–3), 291–298. 10.1016/j.jad.2008.03.013 18471893

[bibr146-10870547251341597] Thapar A. Collishaw S. Pine D. S. Thapar A. K. (2012). Depression in adolescence. The Lancet, 379(9820), 1056–1067. 10.1016/S0140-6736(11)60871-4 PMC3488279 22305766

[bibr147-10870547251341597] Thapar A. Livingston L. A. Eyre O. Riglin L. (2023). Practitioner review: Attention-deficit hyperactivity disorder and autism spectrum disorder: The importance of depression. Journal of Child Psychology and Psychiatry, 64(1), 4–15. 10.1111/jcpp.13678 35972029 PMC10087979

[bibr148-10870547251341597] Tiego J. Martin E. A. DeYoung C. G. Hagan K. Cooper S. E. Pasion R. Satchell L. Shackman A. J. Bellgrove M. A. Fornito A. , & the HiTOP Neurobiological Foundation Work Group. (2023). Precision behavioral phenotyping as a strategy for uncovering the biological correlates of psychopathology. Nature Mental Health, 1(5), 304–315. 10.1038/s44220-023-00057-5 37251494 PMC10210256

[bibr149-10870547251341597] Turgay A. (1994). Disruptive behavior disorders child and adolescent screening and rating scales for children, adolescents, parents and teachers. Integrative Therapy Institute Publication.

[bibr150-10870547251341597] Turgay A. Ansari R. (2006). Major depression with ADHD: In children and adolescents. Psychiatry (Edgmont (Pa.: Township)), 3(4), 20–32. 21103168 PMC2990565

[bibr151-10870547251341597] Ulloa R. E. Ortiz S. Higuera F. Nogales I. Fresán A. Apiquian R. Cortés J. Arechavaleta B. Foulliux C. Martínez P. Hernández L. Domínguez E. de la Peña F. (2006). Estudio de fiabilidad interevaluador de la versión en español de la entrevista Schedule for affective disorders and schizophrenia for school-age children—present and lifetime version (K-SADS-PL) [Interrater reliability of the Spanish version of schedule for affective disorders and schizophrenia for school-age children—present and lifetime version (K-SADS-PL)]. Actas Espanolas de Psiquiatria, 34(1), 36–40. 16525903

[bibr152-10870547251341597] Vacher C. Goujon A. Romo L. Purper-Ouakil D. (2020). Efficacy of psychosocial interventions for children with ADHD and emotion dysregulation: A systematic review. Psychiatry Research, 291, Article 113151. 10.1016/j.psychres.2020.113151 32619822

[bibr153-10870547251341597] Vares E. A. Salum G. A. Spanemberg L. Caldieraro M. A. Fleck M. P. (2015). Depression dimensions: Integrating clinical signs and symptoms from the perspectives of clinicians and patients. PLoS ONE, 10(8), e0136037. 10.1371/journal.pone.0136037 PMC4552383 26313556

[bibr154-10870547251341597] Vassilopoulos A. Nicholl M. Wolf R. M. Slifer K. J. Cirincione L. (2020). Discrepancies in assessing symptoms of depression in adolescents with diabetes using the patient health questionnaire and semi-structured interviews. Diabetes Spectrum, 33(4), 339–346. 10.2337/ds20-0010 33223772 PMC7666605

[bibr155-10870547251341597] Wechsler D. (1991). Wechsler intelligence scale for children (3rd ed.). (WISC-III): Manual. Psychological Corporation.

[bibr156-10870547251341597] Wechsler D. (1997). WAIS-III administration and scoring manual. Psychological Corporation.

[bibr157-10870547251341597] Wechsler D. (2004). Wechsler intelligence scale for children – fourth UK edition (WISC-IV UK) ( Marshall L. A. Coyne I. , Eds.). British Psychological Society. 10.53841/bpstest.2004.wisc4

[bibr158-10870547251341597] Wechsler D. (2011). Wechsler abbreviated scale of intelligence (2nd ed.). PsycTESTS Dataset.

[bibr159-10870547251341597] Weller E. B. Weller R. A. Fristad M. A. Rooney M. T. Schecter J. (2000). Children’s Interview for Psychiatric Syndromes (ChIPS). Journal of the American Academy of Child & Adolescent Psychiatry, 39(1), 76–84. 10.1097/00004583-200001000-00019 10638070

[bibr160-10870547251341597] Weller E. B. Weller R. A. Rooney M. T. Fristad M. A. (1999). Children’s interview for psychiatric syndromes-parent version. American Psychiatric Association.

[bibr161-10870547251341597] Wigham S. Barton S. Parr J. R. Rodgers J. (2017). A systematic review of the rates of depression in children and adults with high-functioning autism spectrum disorder. Journal of Mental Health Research in Intellectual Disabilities, 10(4), 267–287. 10.1080/19315864.2017.1299267

[bibr162-10870547251341597] Williams S. B. O’Connor E. A. Eder M. Whitlock E. P. (2009). Screening for child and adolescent depression in primary care settings: A systematic evidence review for the US preventive services task force. Pediatrics, 123(4), e716–e735. 10.1542/peds.2008-2415 19336361

[bibr163-10870547251341597] Wolraich M. L. Feurer I. D. Hannah J. N. Baumgaertel A. Pinnock T. Y. (1998). Obtaining systematic teacher reports of disruptive behavior disorders utilizing DSM-IV. Journal of Abnormal Child Psychology, 26(2), 141–152. 10.1023/A:1022673906401 9634136

[bibr164-10870547251341597] Wolraich M. L. Lambert W. Doffing M. A. Bickman L. Simmons Worley K. (2003). Psychometric properties of the Vanderbilt ADHD diagnostic parent rating scale in a referred population. Journal of Pediatric Psychology, 28(8), 559–568. 10.1093/jpepsy/jsg046 14602846

[bibr165-10870547251341597] World Health Organization. (1992). The ICD-10 classification of mental and behavioural disorders: Clinical descriptions and diagnostic guidelines.

[bibr166-10870547251341597] Xia W. Shen L. Zhang J. (2015). Comorbid anxiety and depression in school-aged children with attention deficit hyperactivity disorder (ADHD) and selfreported symptoms of ADHD, anxiety, and depression among parents of school-aged children with and without ADHD. Shanghai Arch Psychiatry, 27(6), 356–367. 10.11919/j.issn.1002-0829.215115 27199527 PMC4858507

[bibr167-10870547251341597] Yoshimasu K. Barbaresi W. Colligan R. Voigt R. Killian J. Weaver A. Katusic S. (2012). Childhood ADHD is strongly associated with a broad range of psychiatric disorders during adolescence: A population-based birth cohort study. Journal of Child Psychology and Psychiatry, 53(10), 1036–1043. 10.1111/j.1469-7610.2012.02567.x 22647074 PMC3608464

[bibr168-10870547251341597] Young S. Adamo N. Ásgeirsdóttir B. B. Branney P. Beckett M. Colley W. Cubbin S. Deeley Q. Farrag E. Gudjonsson G. Hill P. Hollingdale J. Kilic O. Lloyd T. Mason P. Paliokosta E. Perecherla S. Sedgwick J. Skirrow C. Tierney K. van Rensburg K. Woodhouse E. (2020). Females with ADHD: An expert consensus statement taking a lifespan approach providing guidance for the identification and treatment of attention-deficit/ hyperactivity disorder in girls and women. BMC Psychiatry, 20(1), 404. 10.1186/s12888-020-02707-9 32787804 PMC7422602

[bibr169-10870547251341597] Yüce M. Zoroglu S. S. Ceylan M. F. Kandemir H. Karabekiroglu K. (2013). Psychiatric comorbidity distribution and diversities in children and adolescents with attention deficit/hyperactivity disorder: A study from Turkey. Neuropsychiatric Disease and Treatment, 9, 1791. 10.2147/NDT.S54283 24265552 PMC3833407

[bibr170-10870547251341597] Zahid S. Bodicherla K. P. Eskander N. Patel R. S. (2020). Attention-Deficit/Hyperactivity Disorder and suicidal risk in major depression: Analysis of 141,530 adolescent hospitalizations. Cureus, 12, e7949. 10.7759/cureus.7949 PMC7270943 32509475

[bibr171-10870547251341597] Zerón-Rugerio M. F. Carpio-Arias T. V. Ferreira-García E. Díez-Noguera A. Cambras T. Alda J. Á. Izquierdo-Pulido M. (2021). ADHD subtypes are associated differently with circadian rhythms of motor activity, sleep disturbances, and body mass index in children and adolescents: A case–control study. European Child & Adolescent Psychiatry, 30(12), 1917–1927. 10.1007/s00787-020-01659-5 33063173

[bibr172-10870547251341597] Zimmerman M. Galione J. N. Chelminski I. McGlinchey J. B. Young D. Dalrymple K. Ruggero C. J. Witt C. F. (2010). A simpler definition of major depressive disorder. Psychological Medicine, 40(3), 451–457. 10.1017/S0033291709990572 19627639

